# Construction of MUC2 promoter-type periodontitis DNA vaccine candidate plasmid and preliminary exploration of the effect of *Lactobacillus rhamnosus* on its expression

**DOI:** 10.1371/journal.pone.0353062

**Published:** 2026-07-16

**Authors:** Bin Chen, Rujia Wang, Yongfeng Zhao, Yufei Qian, Guanghang Wang, Qin Fan, Guohui Bai

**Affiliations:** 1 Key Laboratory of Oral Disease Research, School of Stomatology, Zunyi Medical University, Zunyi, China; 2 Zunyi Key Laboratory of Oral Disease Research, School of Stomatology, Zunyi Medical University, Zunyi, China; 3 Hospital of Stomatology, Zunyi Medical University, Zunyi, China; 4 First People’s Hospital, Kunming, China; 5 Fifth Affiliated Hospital of Zunyi Medical University, Zhuhai, China; Texas A&M University, UNITED STATES OF AMERICA

## Abstract

**Background:**

Conventional cytomegalovirus (CMV) promoter-type periodontitis vaccines can offer protection against periodontitis; however, their antigen genes can be widely expressed in the body, resulting in significant toxic effects. This study aimed to modify the CMV promoter for targeted expression of antigen genes, investigate appropriate methods for regulating its expression, and explore its regulatory mechanism.

**Methods:**

We searched for the MUC2 promoter sequence in the UCSC database, replaced the original broad-spectrum CMV promoter type pVAX1-CMV_pro_-*HA2-fimA* periodontitis DNA vaccine candidate with the MUC2 promoter sequence, and constructed the pVAX1-MUC2_pro_*-HA2-fimA-EGFP* periodontitis DNA vaccine. We assessed the intestinal-specific expression ability of the MUC2 promoter-type periodontitis DNA vaccine candidate by transfecting different tissue cells. Subsequently, various concentrations of *Lactobacillus rhamnosus* supernatant were used to stimulate intestinal tissue cells transfected with the MUC2pro periodontitis DNA vaccine. Real-time quantitative polymerase chain reaction (RT-qPCR) and Western blotting (WB) assays were used to confirm the regulatory effect of *Lactobacillus rhamnosus* supernatant. After transfection, TGF-β pathway inhibitors were used to target intestinal tissue cells cultured with *Lactobacillus rhamnosus* supernatant. RT-qPCR and WB assays were used to assess the impact of TGF-β inhibitors on the regulatory effect of *Lactobacillus rhamnosus* supernatant. All statistical analyses were performed using SPSS 29.0, with one-way ANOVA for multiple group comparisons and LSD/Tamhane’s T2 for post-hoc tests; *P* < *0.05* was considered statistically significant.

**Results:**

After transfection with the MUC2 promoter-type periodontitis vaccine plasmid, green fluorescence was exclusively detected in LS-174T cells, while it was absent in LX-2, BEAS-2B, and U251 cells. Compared with the control group of LS 174T cells transfected without SN, the 2% SN group exhibited no enhancement of vaccine antigen expression (*P* > 0.05). The 4% SN group enhanced vaccine antigen expression (*P* < 0.05), while the 8% SN group significantly promoted vaccine antigen expression (*P* < 0.01). Compared with the control group without SN, the PFD group exhibited no inhibitory effect on vaccine antigen expression in transfected LS-174T cells (*P* > 0.05). Compared with the 8% SN group, the expression of vaccine antigens in LS-174T cells transfected with the 8% SN + PFD group was significantly inhibited (*P* < 0.05).

**Conclusions:**

Periodontitis DNA vaccine candidate plasmid pVAX1-MUC2_pro_-*HA2-fimA-EGFP* was engineered for intestinal tissue-specific expression. Additionally, an appropriate concentration of *Lactobacillus rhamnosus* supernatant enhances MUC2 promoter-type periodontitis DNA vaccine candidate expression, which may be associated with TGF-β pathway regulation. EGFP was used as a surrogate marker for HA2/fimA expression in this study, and P2A cleavage efficiency was assumed but not validated, representing an indirect readout of antigen expression.

## Introduction

Periodontitis is a chronic infectious disease primarily caused by bacterial biofilm in the mouth, resulting in irreversible damage to the supporting tissues surrounding the teeth. The typical symptoms of periodontitis include gingival inflammation, periodontal pocket formation, alveolar bone absorption, and tooth loosening and displacement. In severe cases, it may result in tooth loss, closely associated with chronic diseases, including diabetes and cardiovascular disease [1]. The conventional periodontal treatment methods primarily include the removal of dental plaque and calculus, drug therapy, and surgical treatment in severe cases. Although these methods can partially regulate the progression of periodontitis, they are usually repeated and often fail to eradicate pathogens, complicating the prevention of periodontitis recurrence. Additionally, the mechanical cleaning procedure may cause injury to gingival tissue, while drug therapy may result in drug resistance [2]. Therefore, new treatment strategies for periodontitis have emerged as a prominent research focus.

DNA vaccine candidate (nucleic acid vaccine) is an immunobiological agent that stimulates the body to generate protective antibodies, thereby eliciting specific protective effects against specific diseases [3,4]. A previous study reported that *Porphyromonas gingivalis* (*P. gingivalis)* is a major pathogen responsible for chronic periodontitis. The selection of DNA vaccine candidate antigens for periodontitis primarily concentrates on the expansion of the virulence factor of gingivalis [5]. The fimA is a significant factor in adhesion, and the essential structural domain of gingival (HagA) implicated in *P.* *gingivalis* has a coagulative effect [6]. The HagA coding region comprises four segments: HA1, HA2, HA3, and HA4. HA2 is the key region of *P. gingivalis*. HA2 regulates and mediates bacterial aggregation and lysis of red blood cells, adhesion to periodontal tissue, and the degradation of periodontal tissue morphology. We prepared a periodontitis DNA vaccine candidate pVAX1-CMV_pro_-*HA2-fimA* using the cytomegalovirus promoter (CMV_pro)_ to drive the *HA2* and *fimA* antigen genes in the early stage. The target genes *HA2* and *fimA* can be accurately expressed, exhibiting specific therapeutic effects on experimental periodontitis in rats. However, as the research progresses, certain issues are increasingly revealed and require immediate resolution. The cytomegalovirus (CMV) promoter induces target gene expression; however, it lacks specificity, leading to widespread antigen protein expression across different tissues and difficulty in regulating expression levels [7,8]. Promoters, as essential cis-regulatory elements in gene expression, are essential in gene engineering expression vectors and play a significant role in the regulation of gene expression [9,10]. Therefore, the investigation and development of promoter functional diversity are particularly crucial.

The main solution to the widespread expression and inadequate targeting of DNA vaccine candidates is the development of tissue-specific promoters. Genes regulated by tissue-specific promoters are transcribed or expressed effectively in specific organs or tissues, while their expression in other tissues is infrequent or absent. This promoter sequence comprises several unique cis-acting elements crucial for establishing tissue-specific expression activity. Numerous tissue-specific promoters have been identified across different organisms. This includes specific promoters found in plants, including those targeting vascular tissue (such as the grp1.8 promoter [11], 4CL1 promoter [12]) and floral organ-specific promoters (tobacco TA29 gene promoter [13], g10 gene promoter [14]); In animals, this includes mammary gland-specific promoters (BLG gene promoters [15]), skeletal muscle-specific promoters (including pig alpha-actin gene promoters [16]), and intestinal tissue-specific promoters (IFABP promoters [17], MUC2 promoters [18]). Tissue-specific promoters facilitate the expression of target antigens exclusively in specific organs or tissues, thereby minimizing energy expenditure and biochemical toxicity associated with systemic expression and alleviating the overall physiological burden [19].

The MUC2 promoter is a tissue-specific promoter in the intestine, characterized by extensive blood circulation, numerous secretory cells involved in immune responses, and abundant lymphoid tissue [19]. Therefore, it is regarded as a potential immune target organ that facilitates target gene expression in the intestine, thereby enhancing the production of numerous antibodies in the body. Ogawa et al. [20] further confirmed the potential of intestinal immunotherapy for periodontitis: they performed oral administration experiments of vaccines targeting periodontitis pathogens in mice. The experiments revealed that significant specific antibody responses against the antigen were observed in the serum and saliva of mice immunized with oral vaccines. The secretion of immunoglobulin A (SIgA) and antibody levels in saliva exhibited better results than subcutaneous and intramuscular injection administration methods. Many intestinal-specific proteins, including fatty acid binding proteins, mucins, and villin, are expressed in the intestine [21–23] for specific functions and pathological conditions of the intestine. The specific expression of these proteins depends on the regulation of their corresponding specific promoters. Early studies, including those of Gambus et al. [24] first identified the MUC2 promoter through *in vitro* experiments and confirmed its expression in specific cells and tissues. Furthermore, Gum et al. [25] cloned the MUC2 promoter for the first time using a transgenic mouse model, demonstrating that the reporter gene guided by it can be expressed explicitly in intestinal goblet cells. Additionally, Guan Lizheng et al. developed a specific expression vector using the MUC2 promoter and achieved the specific expression of the β-glucanase gene in mouse intestinal tissue. The MUC2 promoter exhibits more prominent advantages and broader application prospects compared with other intestinal-specific promoters. Based on previous research findings, preparing a periodontitis vaccine that exclusively expresses the target antigen in the intestine is possible.

*Lactobacillus rhamnosus* is a Gram-positive lactobacillus that can survive in low pH, high bile salts, and anaerobic environments in the gastrointestinal tract. Extensive research has been conducted on applying *Lactobacillus rhamnosus* in oral health. Glavina et al. [26] reported that yogurt containing *Lactobacillus rhamnosus* can effectively inhibit *Streptococcus mutans* in the oral cavity and has potential anti-caries effects. Gatej et al. [27] reported that *Lactobacillus rhamnosus* can positively affect experimental periodontitis in mice, regardless of the method of administration. A previous study [28] reported that the supernatant of *Lactobacillus rhamnosus* can promote intestinal MUC2 secretion, thereby inhibiting the adhesion of *Escherichia coli* to intestinal epithelial cells and protecting the intestine. Researchers have examined the mechanisms through which *Lactobacillus acidophilus* 1.1859 and 1.2686, *Lactobacillus casei* 1.2435 and 1.539, *Lactobacillus plantarum* TH1 and their lysates and supernatants promote MUC2 mucin expression. The research revealed that *Lactobacillus* can regulate the transcriptional activity of the MUC2 promoter through the TGF-β signaling pathway, thereby regulating MUC2 mucin expression. Therefore, this study explored whether the regulatory effect of Lactobacillus rhamnosus supernatant on MUC2 promoter activity may involve the TGF-β pathway, thereby potentially influencing the expression of the MUC2-type periodontitis vaccine antigen.

This study aimed to construct a MUC2 promoter-type periodontitis DNA vaccine candidate based on the pVAX1-CMV_pro_-*HA2-fimA* periodontitis DNA vaccine candidate already constructed by the research group, combined with the intestinal tissue-specific expression of the MUC2 promoter, replacing the broad-spectrum expression of the CMV promoter on the original vector. We initially confirmed that Lactobacillus rhamnosus supernatant can regulate the transcription activity of the MUC2 promoter and may involve the TGF-β pathway, thereby potentially influencing the expression of the intestinal tissue-specific periodontitis DNA vaccine candidate. A limitation of this study is the use of LS-174T cells (a colon adenocarcinoma line) instead of normal intestinal epithelial cells, which may introduce cancer-associated transcriptional regulation bias on promoter activity. The edibility, excellent intestinal cell adhesion ability, and acid and bile salt resistance of *Lactobacillus rhamnosus* should be combined with the appropriate amount of the MUC2 promoter type periodontitis DNA vaccine candidate to achieve a specific level of expression regulation of the periodontitis DNA vaccine candidate, increase the immune efficacy and safety of the vaccine, and lay a foundation for its application and promotion.

## Materials and methods

### Construction of plasmids pVAX1-MUC2_pro_-*HA2-fimA-EGFP*

We have commissioned Heyuan Biotechnology (Shanghai) Co., Ltd. to substitute the CMV promoter with the MUC2 promoter in the pVAX1-CMVpro-*HA2-fimA* plasmid utilized by our research group, resulting in the development of the MUC2 promoter type periodontitis DNA vaccine candidate pVAX1-MUC2pro-*HA2-fimA.* We connected an EGFP green fluorescent protein tag through a P2A self-cleaving peptide segment to enhance the detection of target gene expression, as there are no commercially available antibodies for HA2 and fimA. A P2A self-cleaving peptide was inserted to link HA2-fimA and EGFP. Effective P2A cleavage and co-expression of antigen and EGFP were assumed in this study but not directly validated. EGFP was therefore used as an indirect readout for antigen expression [29]. EGFP tags can be removed for independent expression without residues, allowing us to indirectly assess the expression level of vaccine antigens by measuring EGFP green fluorescent protein expression. The plasmid host bacteria are *Escherichia coli*, and the bacterial solution was preserved in a –80 °C refrigerator. The construction processes of the MUC2 promoter-type periodontitis DNA vaccine candidate pVAX1-MUC2_pro_-*HA2-fimA-EGFP* are divided into two steps. The first step is the construction of the pVAX1-MUC2_pro_-*HA2-fimA* plasmid.

### Cell culture and fluorescence observation after transfection

Human hepatic stellate LX-2 cells, human lung epithelial BEAS-2B cells, and human glioma U251 cells were cultured in high glucose DMEM medium containing 10% FBS at 37 °C, 5% CO_2_, with periodic digestion passages to maintain a cell confluence of 70%–80%. Human colon adenocarcinoma LS-174T cells were cultured in MEM (ATCC-modified) medium containing 10% FBS at 37 °C and 5% CO_2_ and maintained cell confluence between 70% and 80% during digestion and passage.

Following digestion of LX-2, BEAS-2B, U251, and LS-174T cells in optimal condition with trypsin, they were uniformly spread in a 6-well plate; upon reaching a cell density of 70%–80%, the pVAX1-MUC2_pro_-*HA2-fimA-EGFP* plasmid was transfected, while the pVAX1-CMV_pro_-*HA2-fimA-EGFP* plasmid served as a control. The transfection experiment was performed with three biological replicates for each cell type, and the fluorescence was observed under a microscope after 36 h.

### Culture and supernatant preparation of *Lactobacillus rhamnosus*

Freeze-dried powder of *Lactobacillus rhamnosus* was procured from Beijing Beina Chuanglian Biotechnology Research Institute. A glass test tube containing 5 mL of high-pressure sterilized MRS broth medium was prepared according to the recovery instructions. Additionally, 0.5 mL of MRS broth medium was extracted and injected into the freeze-dried tube. After complete dissolution, it was returned to the liquid test tube. Subsequently, it was converted into a bacterial suspension. A line was drawn on a solid plate and subsequently inverted in an anaerobic biochemical incubator at 37°C for 24h. We selected a single colony of *Lactobacillus rhamnosus*, previously activated for two generations, and inoculated it into a high-pressure sterilized MRS broth liquid culture medium. Furthermore, it was incubated under constant temperature conditions of 37°C, and we measured the absorbance of the bacterial solution at intervals. When the OD_600_ value reached 0.5, the bacterial population achieved the optimal density. We stopped the cultivation process to prevent excessive growth that may result in nutrient depletion or accumulation of cellular metabolites. Subsequently, we centrifuged the culture medium at 6,000 rpm for 20 min and filtered the supernatant using a 0.22μM pore size filter to obtain the *Lactobacillus rhamnosus* supernatant under sterile conditions.

### Regulation of MUC2 promoter vaccine antigen expression by the supernatant of Lactobacillus rhamnosus

The LS-174T cells from each group were cultured into a 96-well plate with a cell density of 1 × 10^4^ cells per well. Each group comprised five wells, and 100 μL of MEM medium was added to each well for 24 h. Transfection was performed for 6 h when the cell growth density was approximately 70%–80%. Subsequently, cells were treated with 100 μL of SN (SN/MEM medium) at volume fractions of 2%, 4%, 8%, and 16%, respectively. The control group received an equivalent volume of MEM medium without SN. After 36 h of treatment, we removed the 96-well plate. The culture medium containing SN was removed to avoid the influence of SN on absorbance measurement, cleaned twice with basic culture medium, and 100 μL of prepared Cell Counting Kit-8 (CCK-8) solution (CCK-8 and serum-free MEM culture medium prepared in a 1:10 ratio) were added to each well and incubated in a constant temperature cell culture incubator for 1.5h. The absorbance values of CCK-8-treated 96-well plates were determined at 450 nm using a microplate reader.

### Quantitative real-time reverse transcriptase polymerase chain reaction (RT-qPCR)

The LS-174T cells with good growth status were selected, digested, centrifuged, and inoculated into a 6-well plate containing 2mL of complete culture medium at 5 × 10^5^ cells per well. Each group was comprised of three biological replicates. When the cell growth density reached 70%–80%, the cells were transfected according to the instructions of the transfection kit (Lipofectamine 3000 transfection reagent, product number: C10511-05, Thermo Fisher Scientific). After 6 h of transfection, the cell culture medium was replaced with a MEM culture medium containing 0,2%,4%,and 8%SN. The culture plate was maintained in a constant temperature cell culture incubator for 36 h. RT-qPCR was used to detect antigen gene expression in all four groups of the cells following the instructions on the reagent kit (product number: R1200, Beijing Solaibao Technology Co., Ltd.) to extract total RNA from cells. The volume of RNA required for reverse transcription and the volume of the water were calculated based on the measured RNA concentration. EasyScript from Full Gold Company ® One-Step gDNA Removal and cDNA Synthesis SuperMix assay kit, thaw RNA, and reagents stored in a -80°C freezer on ice were used to prepare reverse transcription reaction solution. The mixture was thoroughly mixed to obtain a 20 μL reaction system, and the reverse transcription reaction conditions in the PCR instrument were configured to 42°C, 15 min; 85°C, 5 s; 4°C, terminate. The obtained cDNA product was diluted 10 times and used as a template for subsequent experiments. Primers were designed using Primer Premier 5 software referencing the GenBank database, with GAPDH as the internal control, and synthesized by Shanghai Shenggong Company. Table 1 presents the primer sequences of the target genes. The green qPCR SuperMix premixed reagent kit was configured with three sub-wells for each gene in each sample using PerfectStart from Full Gold Company ®. According to the required reaction conditions in the manual, the CFX CONNECT Real-Time PCR system was used for the real-time PCR reaction, and the reaction system was configured on ice.

**Table 1:** Primer sequences of target genes and internal control.

Gene	Sequence
EGFP	(F)ACCCTCGTGACCACCCTGAC
	(R)TGTAGTTGCCGTCGTCCTTGAAG
GAPDH	(F)CAGGAGGCATTGCTGATGAT
	(R)GAAGGCTGGGGCTCATTT

### Western blotting (WB)

LS-174T cells with good growth status were seeded into 6-well plates at a density of 5 × 10⁵ cells per well (3 biological replicates per group) and transfected with the plasmid when cell confluence reached 70%–80% using Lipofectamine 3000 (Cat. No.: C10511-05, Thermo Fisher Scientific). For SN concentration gradient treatment, the medium was replaced with MEM complete medium containing 0, 2%, 4% or 8% SN at 6h post-transfection; for PFD combined treatment, cells were divided into control, PFD, 8% SN and 8% SN + 32μM PFD groups, with the corresponding medium replacement at 6h post-transfection. All cells were cultured for an additional 36 h, then total cellular protein was extracted by lysing cells with RIPA lysis buffer containing 1 mM PMSF and protease-phosphatase inhibitor cocktail on ice for 20 min, followed by centrifugation at 12,000 rpm for 15 min at 4°C. The protein concentration was determined by BCA assay (Yamei Biotech) at 562 nm, and 30 μg of total protein per sample was denatured with 5 × loading buffer at 100°C for 10 min. Protein separation was performed by 12.5% SDS-PAGE, followed by semi-dry transfer onto methanol-activated PVDF membranes at 25V and 1.3A for 7 min. The membranes were blocked with 5% BSA for 2h at room temperature, then incubated overnight at 4°C with rabbit polyclonal anti-EGFP antibody (1:1000) and rabbit monoclonal anti-β-Actin antibody (1:10000). After TBST washing (3 times, 10 min each), the membranes were incubated with HRP-conjugated secondary antibody (1:2000) for 2 h at room temperature, and protein bands were visualized by ECL chemiluminescence kit. Band intensities were quantified by densitometric analysis using ImageJ 1.8.0 software, normalized to β-actin loading control, and expressed as relative protein levels (fold change ± SD).

### Mechanism of *Lactobacillus rhamnosus* supernatant regulating MUC2 promoter type periodontitis DNA vaccine candidate through TGF-β pathway

#### CCK-8.

Each group of cells was incubated at 1 × 10^4^ cells/well on a 96-well plate, with five wells in each group. 100 μL of culture medium was added to each well and cultured for 24h. When the cell growth density was approximately 70%–80%, the cells were transfected for 6h (transfection steps were the same as those mentioned in section 2.2.2 of the first part). The PFD mother liquor was diluted to 2, 4, 8, 16, 32, 64, and 128μM in complete medium containing 8% L. rhamnosus supernatant (SN). this concentration of 32μM was selected based on preliminary dose-response experiments and previous literature reports [30,31], which confirmed that 32μM PFD is an effective concentration for inhibiting the TGF-β pathway without obvious cytotoxicity. Subsequently, different concentrations of PFD (0, 2, 4, 8, 16, 32, 64, and 128μM) were added and treated for 36 h. The cell viability of each group was detected using CCK-8 assay. A 96-well plate was extracted, and 100 μL of prepared CCK-8 solution (CCK-8 and serum-free MEM prepared using a 1:10 ratio) was added to each well. The cells were incubated in CO_2_ at a constant temperature incubator for 1.5h. The absorbance values of the CCK-8 treated 96-well plates were determined at 450 nm using a microplate reader.

#### Quantitative real-time reverse transcriptase polymerase chain reaction (RT-qPCR).

The cells were grouped into the control group, PFD group, 8% SN group, and PFD + 8% SN group. Additionally,5 × 10^5^ LS-174T cells were incubated per well into a 6-well plate containing an appropriate complete culture medium, with three biological replicates in each group. When the cell growth density reached 70%–80%, we performed cell transfection according to the instructions of the transfection kit (Lipofectamine 3000 transfection reagent, product number: C10511-05, Thermo Fisher Scientific). After transfection for 6 h, the culture medium was replaced with MEM complete culture medium, MEM complete culture medium+32μM PFD, MEM complete culture medium containing 8% SN, MEM complete culture medium containing 8% SN + 32μM PFD, and processed 36 cells sequentially. The whole cell RNA was extracted after hours.

#### WB.

The cells were grouped into the control group, PFD group, 8% SN group, and PFD + 8% SN group. Additionally, 5 × 10^5^LS-174T cells were incubated per well into a 6-well plate containing an appropriate complete culture medium, with three biological replicates in each group. When the cell growth density reached 70%–80%, cell transfection was performed according to the instructions of the transfection kit (Lipofectamine 3000 transfection reagent, product number: C10511-05, Thermo Fisher Scientific). After transfection for 6 h, the culture medium was replaced with MEM complete culture medium, MEM complete culture medium+32μM PFD, MEM complete culture medium containing 8%SN, MEM complete culture medium containing 8% SN + 32μM PFD,and cultured for an additional 36h. The total protein of the cells was extracted after 36 h of culture, and WB was performed according to the method described in section 2.6 (including densitometric quantification and normalization to β-actin).

### Statistical analysis

Statistical analysis was performed using SPSS 29.0 software. Numerical variables following a normal distribution were described as mean ± standard deviation (x̄ ± s). One-way analysis of variance (ANOVA) was used to compare differences among multiple groups. If the variances were homogeneous, pairwise comparisons were conducted using the LSD test; if the variances were heterogeneous, Tamhane’s T2 test was applied. A *P*-value <0.05 was considered statistically significant. All experiments were performed with at least three biological replicates as indicated in each subsection; densitometric analysis of WB bands was performed using ImageJ 1.8.0 software (National Institutes of Health, Bethesda, MD, USA). All in vitro cell experiments performed in this study did not involve human/animal tissue samples, therefore ethical approval was not required.

## Results

### Plasmid construction and specific detection

**Fig 1:**
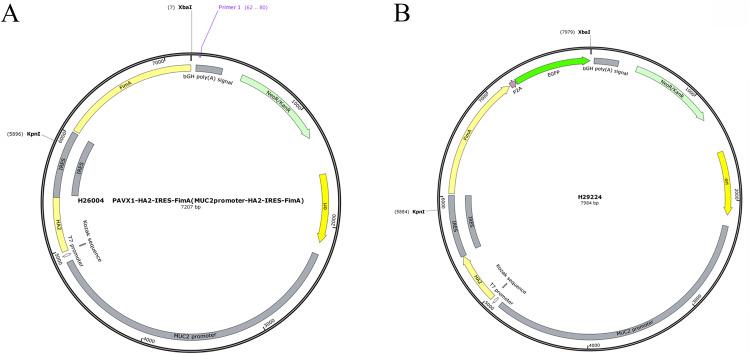
Recombinant plasmid profiles. Note: **(A)** Recombinant plasmid pVAX1-MUC2_pro_*-HA2-fimA* profile. Recombinant plasmid pVAX1-MUC2_pro_*-HA2-fimA-EGFP* profile..

**Fig 2:**
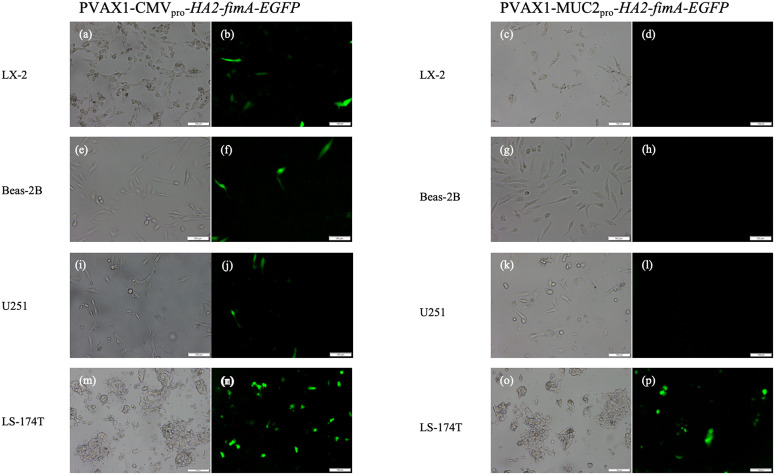
Fluorescence observation results of LX-2, Beas-2B, U251, and LS-174T cells transfected for 36 h (40 × , n = 3 biological replicates). Scale bar = 100 μm. Green fluorescence was only detected in LS-174T cells, indicating intestinal-specific expression of the MUC2 promoter. EGFP expression was used as a surrogate marker for HA2/fimA antigen expression.Error bars represent standard deviation (SD); statistical test: none (morphological observation).

Fig 1 depicts the construction information and pattern diagram of the plasmid. After correct comparison with NCBI, the pVAX1-MUC2pro-*HA2-fimA-EGFP* vector was transfected into LX-2, BEAS-2B, U251, and LS-174T cells, and the pVAX1-CMVpro-*HA2-fimA-EGFP* vector was used as a control. After 36 h, the pVAX1-MUC2pro-*HA2-fimA-EGFP* vector was only expressed in LS-174T cells (Fig 2) and was not expressed in LX-2, BEAS-2B, and U251 cells. This confirmed that the pVAX1-MUC2pro-*HA2-fimA-EGFP* vector prepared in this experiment could be specifically expressed in intestinal cells.Of note, EGFP was used as a surrogate marker for HA2/fimA expression because specific antibodies against HA2 and fimA were not commercially available.

### Effect of Lactobacillus rhamnosus supernatant on vaccines

**Fig 3:**
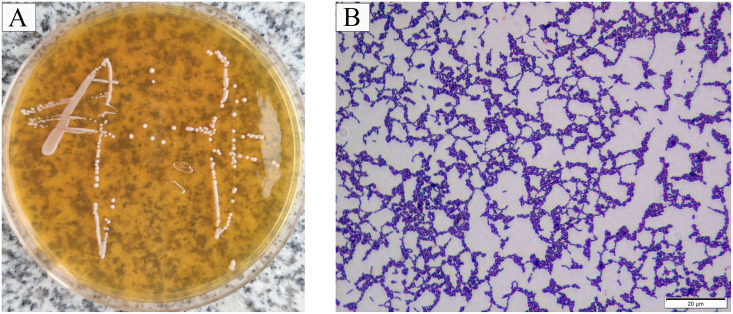
Culture of *Lactobacillus rhamnosus* and Gram staining. **Note:** *Lactobacillus rhamnosus was cultured* in an anaerobic environment (Fig 3A) and Gram-stained (Fig 3B).

**Fig 4:**
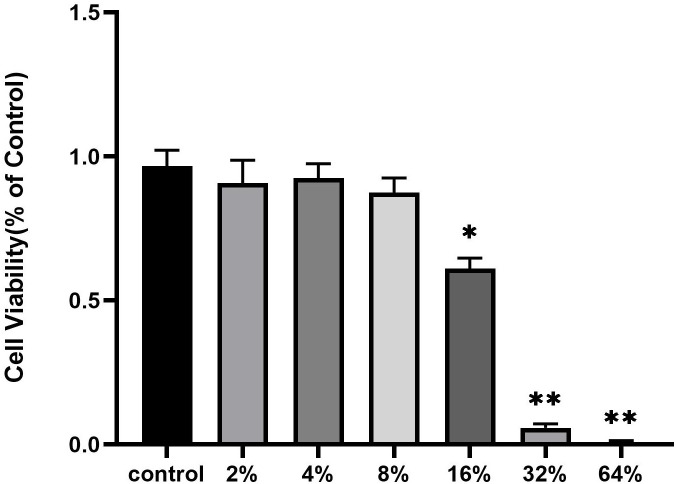
The effect of different concentrations of SN on the activity of transfected LS-174T cells. Note: Data are presented as the mean ± standard deviation (SD) (n = 3 independent experiments). Statistical significance was determined by one-way analysis of variance (ANOVA) followed by LSD post-hoc test. *P < 0.05 and **P < 0.01 versus the control group.

**Fig 5:**
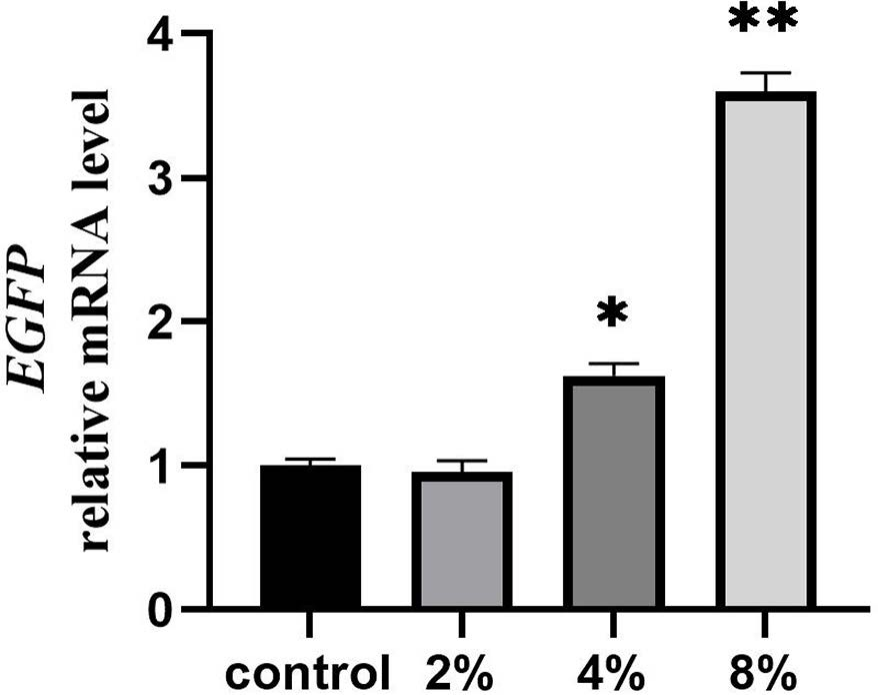
*EGFP* mRNA expression levels in LS-174T cells transfected with SN stimulation. Note:Data are presented as the mean ± SD (n = 3). Statistical analysis was performed using one-way ANOVA with LSD test. *P < 0.05 and **P < 0.01 versus the control group.

**Fig 6:**
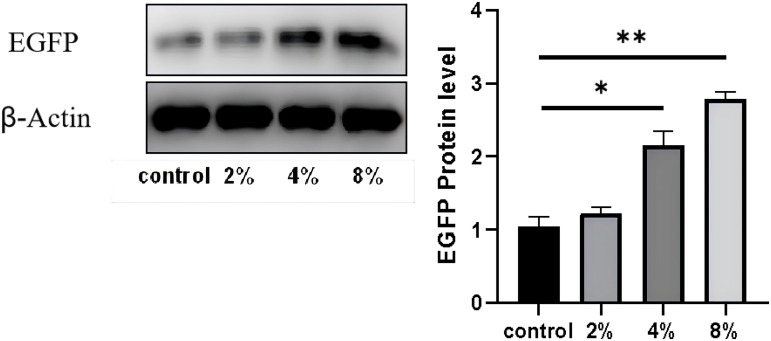
EGFP protein expression levels in LS-174T cells transfected with different concentrations of SN stimulation. Note:Data are presented as the mean ± SD (n = 3). Statistical analysis was performed using one-way ANOVA with LSD test. *P < 0.05 versus the control group.

*Lactobacillus rhamnosus was cultured* in an anaerobic environment (Fig 3) and Gram-stained (Fig 3). Subsequently, it was centrifuged at 6,000 rpm for 20 min, and a 0.22μM pore size filter was used to filter the supernatant. Lactobacillus rhamnosus supernatant was obtained under sterile conditions. Different concentrations of *Lactobacillus rhamnosus* supernatant were used for the experiments (Fig 4), which revealed that 2%, 4%, and 8% concentrations of *Lactobacillus rhamnosus* supernatant have no inhibitory effect on cell viability. Figs 5 and 6 depict that 4% and 8% concentrations of Lactobacillus rhamnosus supernatant promote EGFP expression (4%: *P* < 0.05; 8%: *P* < 0.01 vs control).The effect of TGF-β inhibitor pirfenidone on vaccine expression in the supernatant of *Lactobacillus rhamnosus*

**Fig 7:**
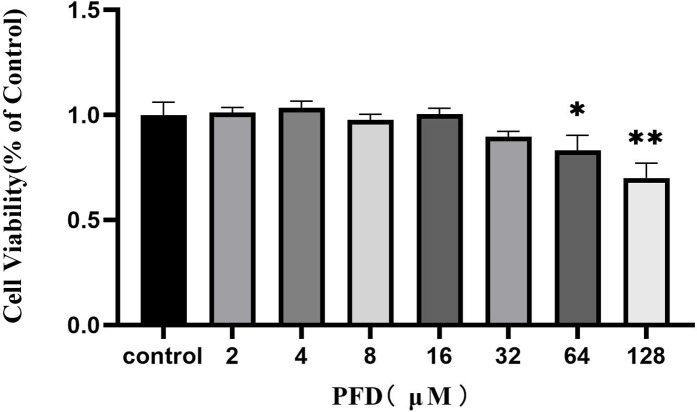
Effect of PFD on the viability of transfected LS-174T cells. Note:Data are presented as mean ± standard deviation (SD) (n = 3 independent experiments). Statistical significance was determined by one-way analysis of variance (ANOVA) followed by LSD post-hoc test, comparing each PFD concentration group to the control group. *P < 0.05 and **P < 0.01 versus the control group.

The CCK-8 assay (Fig 7) revealed that PFD at 2, 4, 8, 16, and 32μM exhibited no inhibitory effect on the viability of LS-174T cells transfected with plasmids, while PFD at 64 and 128μM exhibited a significant inhibitory effect on the viability of LS-174T cells transfected with plasmids. Therefore, the highest concentration of PFD without inhibitory effect on cell viability was selected for subsequent experiments.

**Fig 8:**
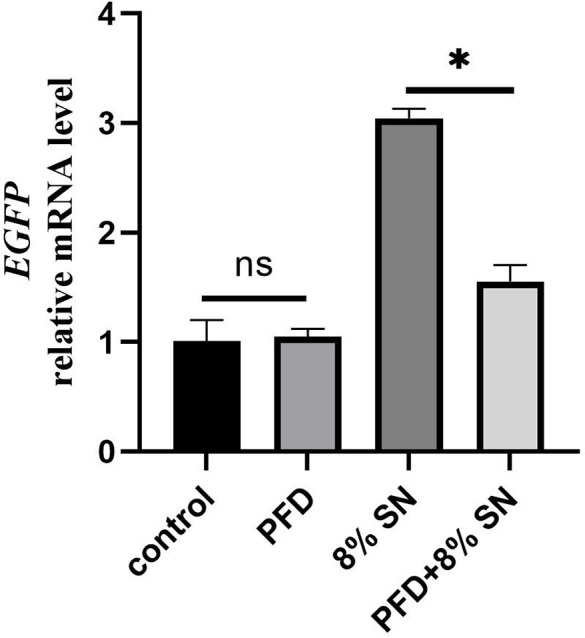
The effect of TGF-β inhibitor PFD on SN-induced *EGFP* mRNA expression. Note:Data are presented as mean ± SD (n = 3). Statistical analysis was performed using one-way ANOVA with LSD test. ns, not significant; *P < 0.05.

**Fig 9:**
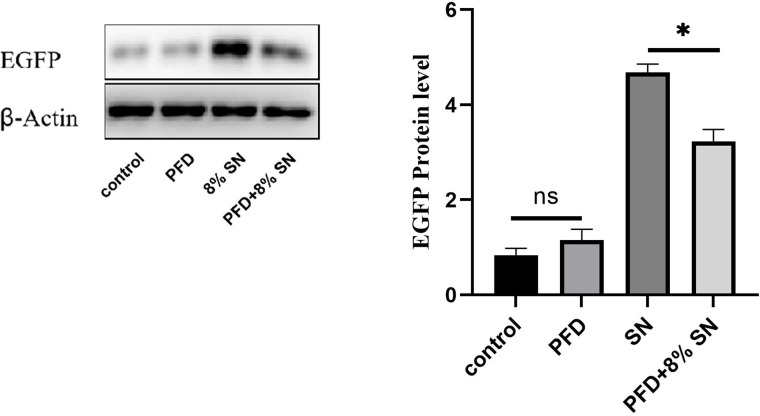
The effect of TGF-β inhibitor PFD on SN-induced EGFP protein expression. Note:The left panel shows representative Western blot images of EGFP and β-Actin (loading control). The right panel shows the quantitative analysis of EGFP protein levels normalized to β-Actin. Data are presented as mean ± SD (n = 3). Statistical analysis was performed using one-way ANOVA with LSD test. ns, not significant; *P < 0.05.

The RT-qPCR (Fig 8) and WB assays (Fig 9) revealed that compared with the control group without SN, the PFD group exhibited no inhibitory effect on vaccine antigen expression in transfected LS-174T cells. Compared with the 8% SN group, the expression of vaccine antigens in LS-174T cells transfected with the 8% SN + PFD group was significantly inhibited (*P* < *0.05*).

## Discussion

Periodontitis is a chronic inflammatory disease that affects the supporting structures of the teeth, with its progression influenced by the interaction between microbial infection and host immune response [32]. The progression of periodontitis is accompanied by a series of pathological processes, with chronic and recurrent inflammatory reactions resulting in gingival erythema, edema, and recession. If the disease progresses unchecked, severe problems may occur, including loose or falling teeth. The treatment of chronic periodontitis primarily involves the removal of accumulated plaque and calculus from the root surface, alleviating the inflammatory response of periodontal tissue, and inhibiting the disease progression. However, a previous study reported that relying solely on mechanical means to remove plaque and dental calculus cannot achieve ideal therapeutic effects [33]. Immunotherapy using periodontitis DNA vaccine candidates has recently received increasing attention in periodontal disease prevention and treatment [34].

In developing DNA vaccine candidates for periodontitis, *P. gingivitis*, the main pathogenic bacterium of chronic periodontitis, has consistently been the research focus. The pathogenic factors of *P. gingivitis* can be primarily divided into two categories: the structural components of the bacterial organism, including pili, cilia, and capsules. Another type is the pathogenic substances secreted by gingivalis and primarily include capsule polysaccharides, lipopolysaccharides, and proteolytic enzymes. In the early stage of the research group, the essential functional region HA2 of the gingivin hemagglutinin gene coding region and the fimA pili protein gene were used as antigen genes to effectively develop the periodontitis DNA vaccine candidate pVAX1-CMVpro-*HA2-fimA*.However, vectors utilizing the CMV promoter from cytomegalovirus have difficulty regulating antigen gene expression within bodily tissues. This study used the MUC2 promoter specific to intestinal tissue to address this issue. The intestinal tissue-specific MUC2 promoter ensures exogenous gene expression exclusively in intestinal tissues, avoiding widespread expression of antigen proteins in the body caused by the CMV promoter. This may potentially reduce the biochemical toxicity and metabolic burden of the vaccine candidate [19], but direct comparative toxicity data between MUC2 and CMV promoter-type vaccine candidates were not obtained in this study. The safety and toxicity of the MUC2 promoter-type vaccine candidate need to be further verified by in vivo experiments.

The plasmid pVAX1-MUC2pro-*HA2-fimA-EGFP*, a DNA vaccine candidate targeting MUC2 promoter-type periodontitis, was developed and constructed. An enhanced green fluorescent protein EGFP tag was added at the end of the plasmid vector to facilitate the observation of the expression effect of the vector. It is a variant of the green fluorescent protein GFP (a luminescent protein derived from jellyfish) that can emit brighter green fluorescence. This green fluorescence exhibits significant stability and superior stress resistance than other fluorescent substances. Compared with other reporter genes, the green fluorescent protein gene does not affect cell growth and division and does not require specific substrate reactions. Its expression can be directly monitored using simple detection equipment, making it an ideal marker for observing exogenous gene expression [35]. The promoter sequence of the MUC2 type intestinal-specific periodontitis vaccine plasmid developed by the research institute is 2614 bp upstream of the 5′ UTR of the MUC2 gene. This sequence includes 73−91 bp upstream of the transcription start site, which influences MUC2 promoter activity, and 171−228 bp upstream of the transcription start site, which governs intestinal-specific expression ability [36]. Additionally, it contains binding sequences for transcription factors, including SP1, AP2, and CDX2 [37]. The MUC2 periodontitis DNA vaccine candidate was transfected into LS174T, LX-2, BEAS-2B, and U251 cells using the liposome transfection method. The results revealed that green fluorescence was exclusively observed in LS174T cells. However, it was absent in other cell lines, demonstrating the intestinal tissue-specific expression capability of the MUC2 periodontitis gene.

Lactic acid is the primary metabolite of Lactobacillus rhamnosus [38], which significantly decreases the pH value of the culture medium containing high concentrations of Lactobacillus rhamnosus supernatant, adversely affecting the viability of LS-174T cells. Therefore, this study assessed the concentration of Lactobacillus rhamnosus supernatant that does not affect cell viability using CCK-8 assays for subsequent experimental studies. The LS-174T cells transfected with MUC2 periodontitis vaccine candidate were treated with different concentrations of Lactobacillus rhamnosus supernatant. RT-qPCR and WB assays revealed that high concentrations of Lactobacillus rhamnosus supernatant could enhance vaccine candidate antigen expressions, indicating that the supernatant of Lactobacillus rhamnosus exerts a regulatory effect on MUC2 periodontitis vaccine candidate expression.

A previous study has reported that the supernatant of Lactobacillus rhamnosus is rich in various active ingredients, including MSP1 (P75), MSP2 (P40), HM0539, and short-chain fatty acids (SCFAs), which are essential in enhancing intestinal barrier capacity and inhibiting various pathogens. Gao et al. [39] reported that the supernatant of Lactobacillus rhamnosus enhances the formation of the intestinal barrier in mice. They isolated the secreted protein HM0539 from the supernatant and discovered that this protein can significantly enhance the protective capacity of the intestinal barrier. The study revealed that HM0539 can enhance intestinal mucosal protein expression and prevent intestinal barrier damage caused by lipopolysaccharide or tumor necrosis factor. SCFAs derived from Lactobacillus rhamnosus metabolites can enhance MUC2 expression, as SCFAs can regulate histone acetylation and methylation within the promoter region of the MUC2 gene, thereby activating the promoter and inducing the secretion of specific mucin MUC2 by goblet cells in the human gut [40]. Butyric acid in SCFAs can specifically identify short-chain fatty acid reaction elements and the cis-acting element AP1 in the MUC2 promoter. This effect enhances promoter activation, facilitating the acetylation of histones H3 and H4 associated with the MUC2 promoter and H3 methylation of the MUC2 promoter, resulting in elevated MUC2 gene expression [41]. Although the supernatant of *Lactobacillus rhamnosus* was confirmed to enhance the expression of the MUC2 promoter-type periodontitis DNA vaccine candidate, the specific bioactive components responsible for this regulatory effect were not identified in this study. The supernatant was only characterized by volume concentration, without standardized detection of metabolite levels (e.g., short-chain fatty acids, HM0539). Additionally, batch variability in bacterial culture (e.g., culture time, temperature) may affect the biological activity of the supernatant, which is a limitation of this study. Isolation and purification of key regulatory factors from the supernatant will be the focus of subsequent research.This study preliminarily investigated whether the supernatant of *Lactobacillus rhamnosus* regulates the intestinal-specific periodontitis vaccine through the TGF-β pathway. The RT qPCR and WB assays revealed that adding only the TGF-β inhibitor PFD to the culture medium did not significantly affect MUC2-type periodontitis DNA vaccine candidate expression. However, adding *Lactobacillus rhamnosus* supernatant to the culture medium can significantly enhance MUC2-type periodontitis DNA vaccine expression. Adding *Lactobacillus rhamnosus* supernatant and TGF-β inhibitor PFD to the culture medium significantly inhibited *Lactobacillus rhamnosus* supernatant promotion effect on MUC2 type periodontitis DNA vaccine candidate expression. These results suggest that the enhancement of MUC2 promoter-driven vaccine expression by L. rhamnosus supernatant may involve the TGF-β pathway; however, direct measurements of TGF-β levels and SMAD2/3 phosphorylation were not performed. It should be noted that the involvement of the TGF-β pathway in this study is inferred primarily from the reversal of the supernatant’s effect by the pharmacological inhibitor PFD. We did not directly measure key pathway components such as TGF-β levels or SMAD2/3 phosphorylation. Therefore, this provides preliminary pharmacological evidence for the pathway’s involvement, and future studies are needed to obtain direct molecular evidence.The TGF-β signaling pathway is an essential pathway in cellular signaling that regulates cell growth, differentiation, migration, apoptosis, and synchronous effects in various biological processes [42]. The TGF-β signaling pathway is essential for maintaining tissue homeostasis and organ development, and it is implicated in disease conditions, including cancer development, fibrosis, and immune regulation. The PFD used in this study is a publicly available TGF-β signaling pathway inhibitor for treating idiopathic pulmonary fibrosis. It can intervene in the mRNA transcription process of TGF-β, reduce TGF-β expression level, and block the downstream signaling pathway of TGF-β [30,31]. Hao Ran et al. reported the significant role of the TGF-β signaling pathway in regulating MUC2 promoter transcriptional activity and MUC2 mucin expression in five strains of lactic acid bacteria, including *Lactobacillus acidophilus* 1.1859 and 1.2686, *Lactobacillus casei* 1.2435 and 1.539, and *Lactobacillus plantarum* TH1.

This study preliminarily confirmed the successful construction of a periodontitis vaccine capable of expressing antigen proteins specifically in the intestine, thereby diminishing the biological toxicity and energy expenditure associated with the conventional CMV promoter-type periodontitis vaccine widely expressed in the body. Besides, this study confirmed the regulatory capacity of *Lactobacillus rhamnosus* supernatant on it. When the vaccine efficacy is insufficient, it can be enhanced by supplementing with *Lactobacillus rhamnosus* dairy products. Antigen protein expression in the vaccine is not necessarily better. The vaccine can be further regulated by regulating the TGF-β pathway, demonstrating its potential for targeted control. This study validated the gut-specific expression ability of the MUC2 type periodontitis DNA vaccine candidate and preliminarily investigated the regulation of MUC2 promoter type periodontitis DNA vaccine expression by *Lactobacillus* rhamnosus supernatant.

Importantly, these results were obtained in LS-174T colon adenocarcinoma cells and cannot be directly generalized to normal intestinal epithelial cells or in vivo vaccine performance. This study was performed in vitro, and in vivo animal studies are required for further validation.Therefore, the present findings are preliminary, and in vivo animal studies are required to further validate the intestinal-specific expression, protective efficacy, and regulatory mechanisms of the MUC2 promoter-based periodontitis vaccine.

## Supporting information

S1 File Uncropped original Western blot images. (ZIP)

S2 File Raw RT-qPCR data of EGFP mRNA. (ZIP)

S3 File Raw CCK-8 cell viability data. (ZIP)

S4 File Original cell fluorescence micrographs. (ZIP)

## Abbreviations

CMV: cytomegalovirus (CMV) promoter
cDNA: complementary DNA
EGFP: Enhanced Green Fluorescent Protein
FimA: Fimbriae A
HA2: Hemagglutinin 2
MUC2: intestinal mucin 2 promoter
PBS: Phosphate buffer solution
*P. gingivalis*: *Porphyromonas gingivalis*
PMSF: Phenylmethylsulfonyl fluoride
PFD: Pirfenidone
RT-qPCR: Real-time quantitative polymerase chain reaction
SIgA: Secretory immunoglobulin A
SN: *Lactobacillus rhamnosus* supernatant
TGF-β: Transforming growth factor-β
