# Supplementary figures and images for: Construction of MUC2 promoter-type periodontitis DNA vaccine candidate plasmid and preliminary exploration of the effect of Lactobacillus rhamnosus on its expression
Source: PLoS One. 2026 Jul 16;21(7):e0353062. doi: 10.1371/journal.pone.0353062 (PMC13375136; doi:10.1371/journal.pone.0353062)

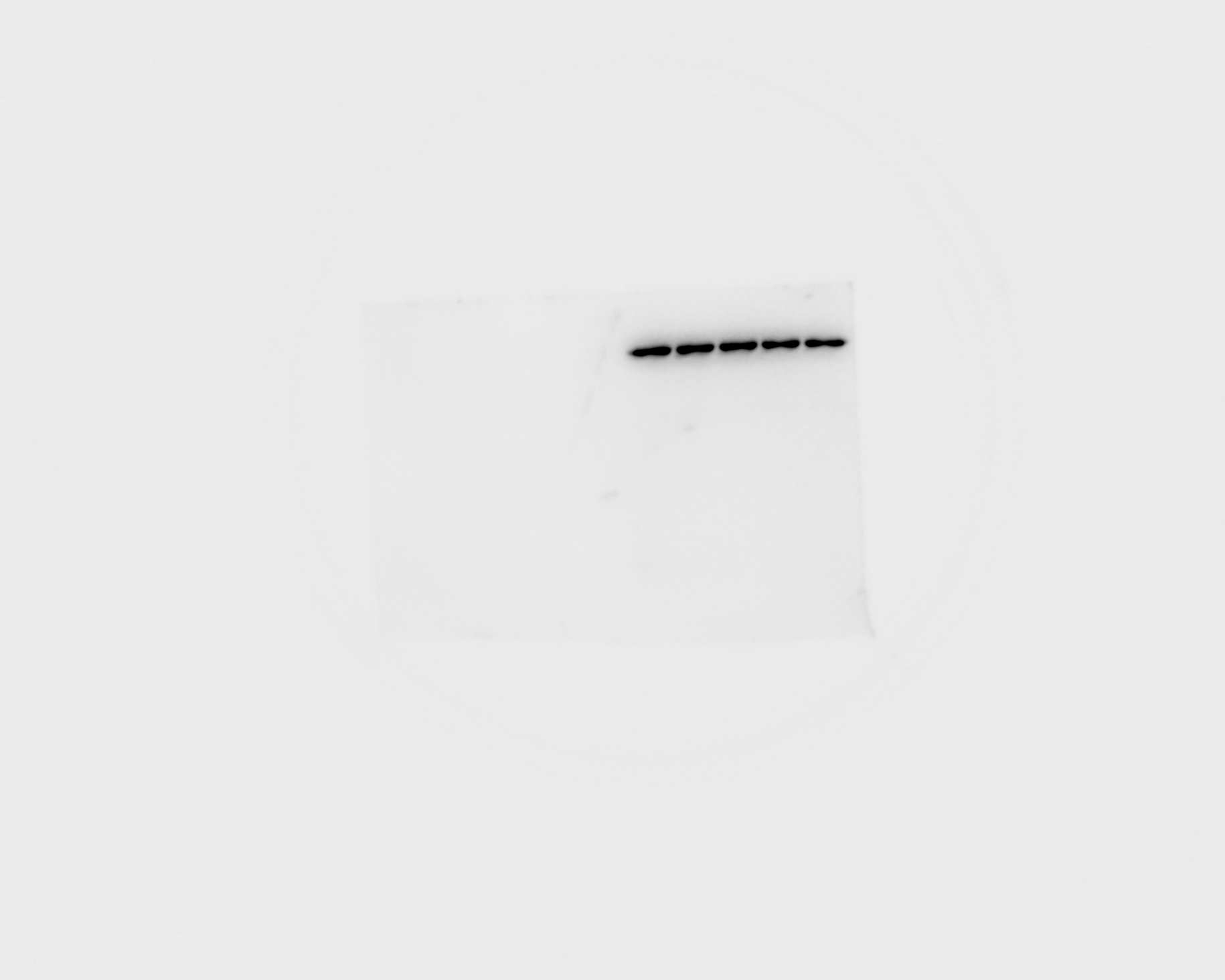

Supplement: S1 File — (ZIP) [file pone.0353062.s001.zip › S1 File. Original uncropped and unadjusted Western blotting images for EGFP and β-actin protein expression/Inhibitor group/zyf b-actin(Chemiluminescence).tif]

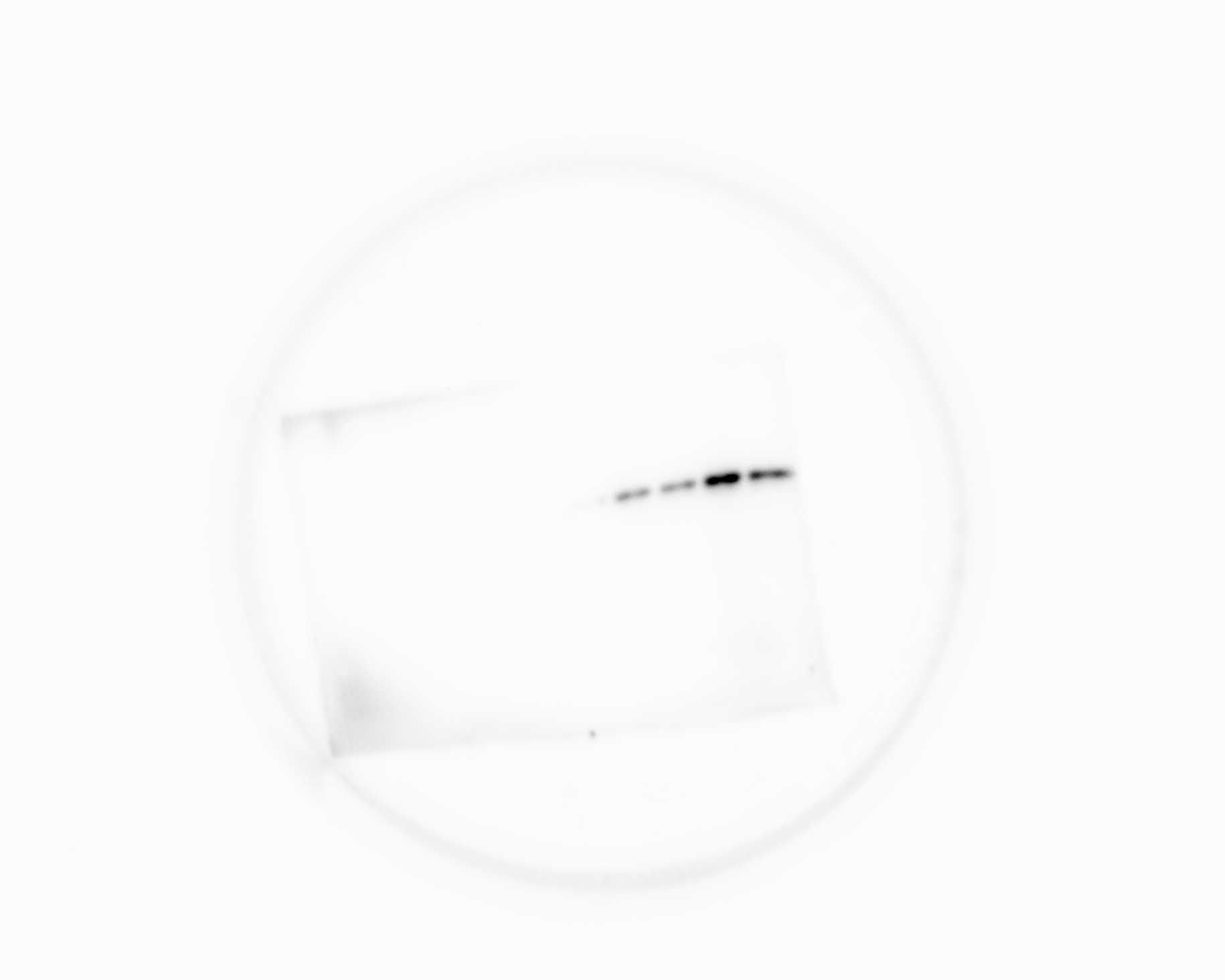

Supplement: S1 File — (ZIP) [file pone.0353062.s001.zip › S1 File. Original uncropped and unadjusted Western blotting images for EGFP and β-actin protein expression/Inhibitor group/zyf EGFP (Chemiluminescence).tif]

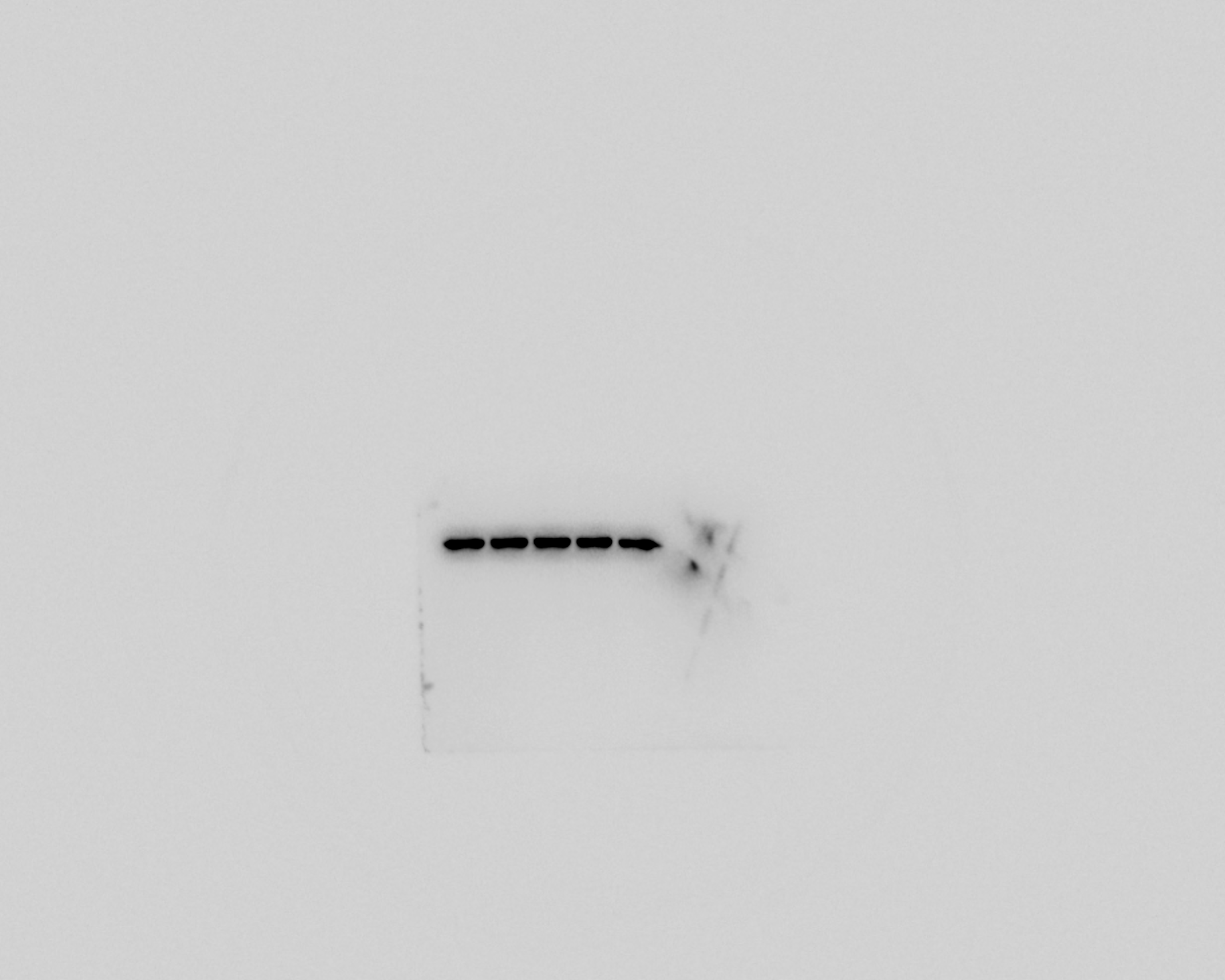

Supplement: S1 File — (ZIP) [file pone.0353062.s001.zip › S1 File. Original uncropped and unadjusted Western blotting images for EGFP and β-actin protein expression/Lactobacillus rhamnosus group/b-actin(Chemiluminescence).tif]

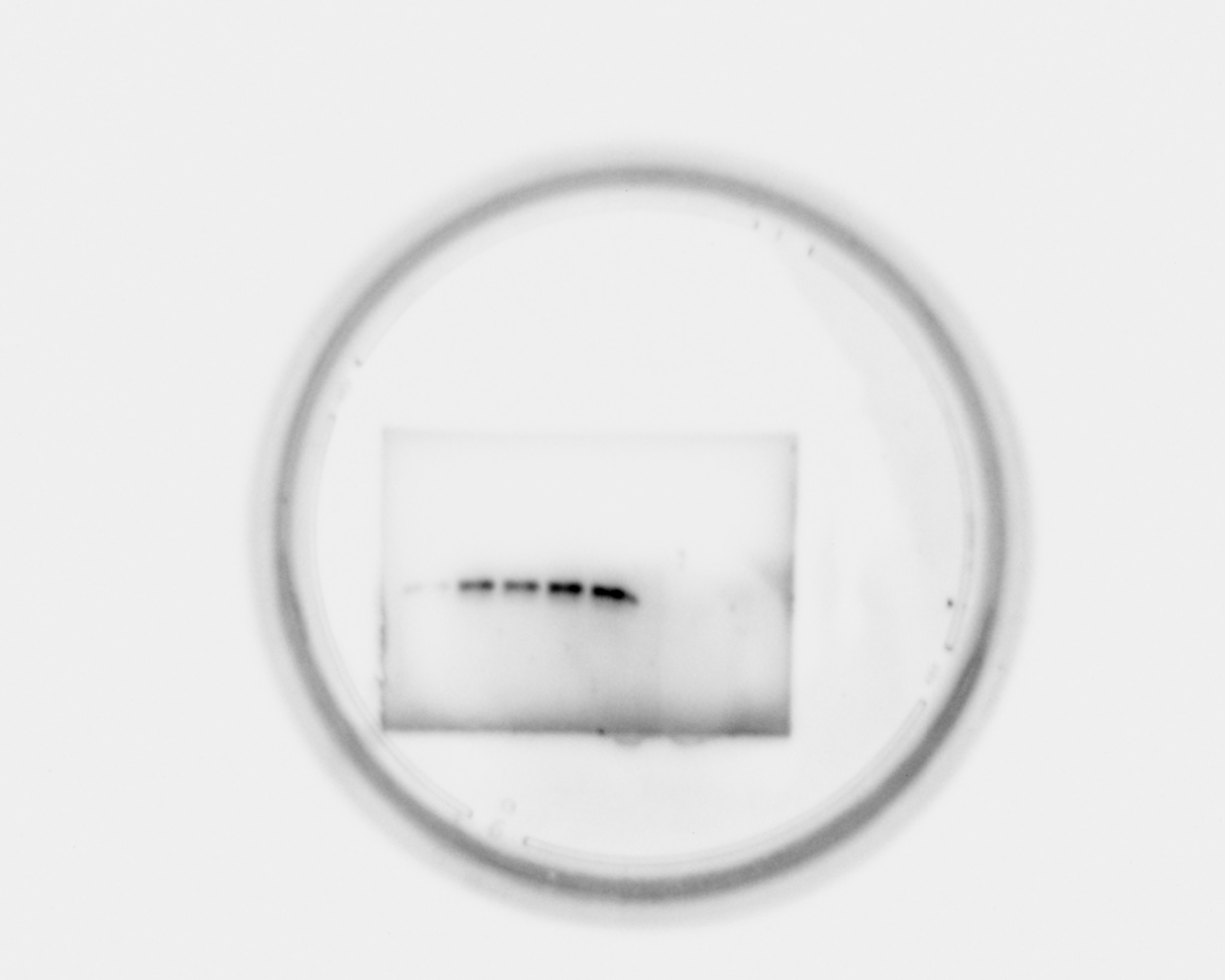

Supplement: S1 File — (ZIP) [file pone.0353062.s001.zip › S1 File. Original uncropped and unadjusted Western blotting images for EGFP and β-actin protein expression/Lactobacillus rhamnosus group/EGFP (Chemiluminescence).tif]

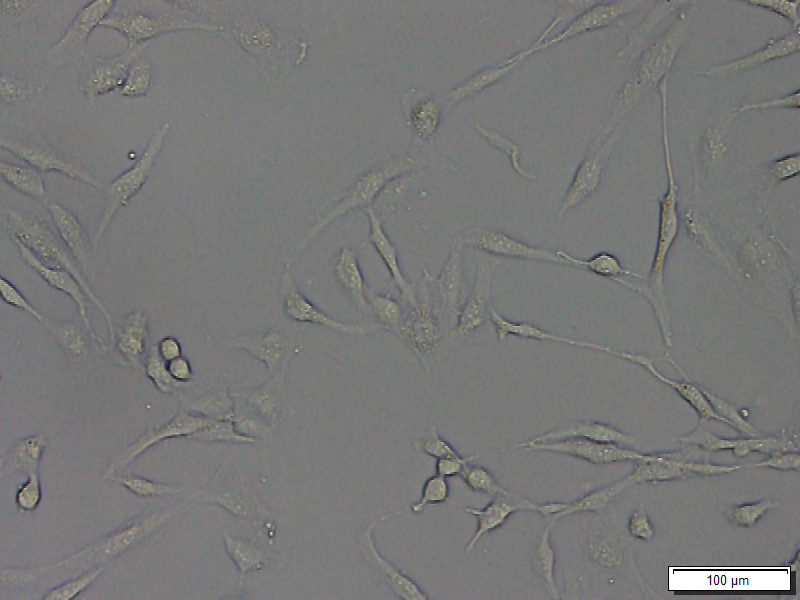

Supplement: S1 File — (ZIP) [file pone.0353062.s001.zip › S4 File. Fluorescent microscopy images of cell samples/beas-2b/Beas-2b cmv 明场.tif]

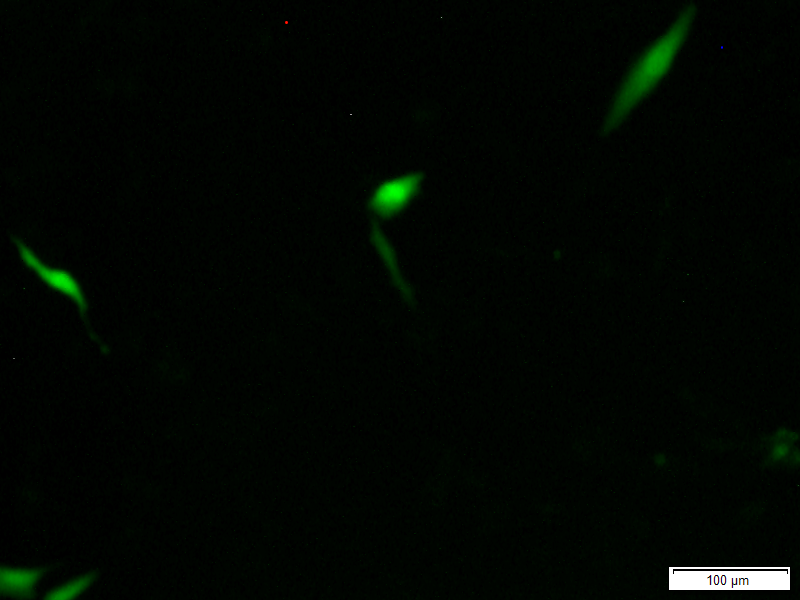

Supplement: S1 File — (ZIP) [file pone.0353062.s001.zip › S4 File. Fluorescent microscopy images of cell samples/beas-2b/Beas-2b cmv 暗场.tif]

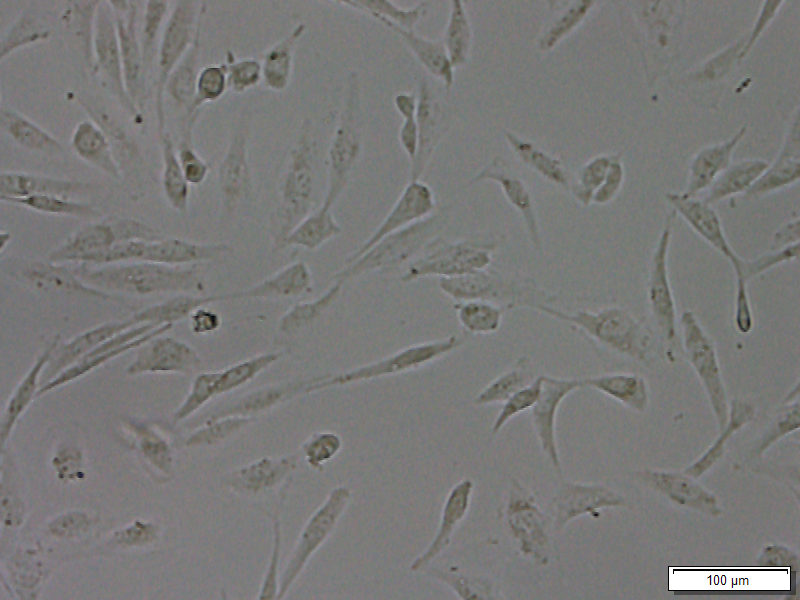

Supplement: S1 File — (ZIP) [file pone.0353062.s001.zip › S4 File. Fluorescent microscopy images of cell samples/beas-2b/Beas-2b muc 明场.tif]

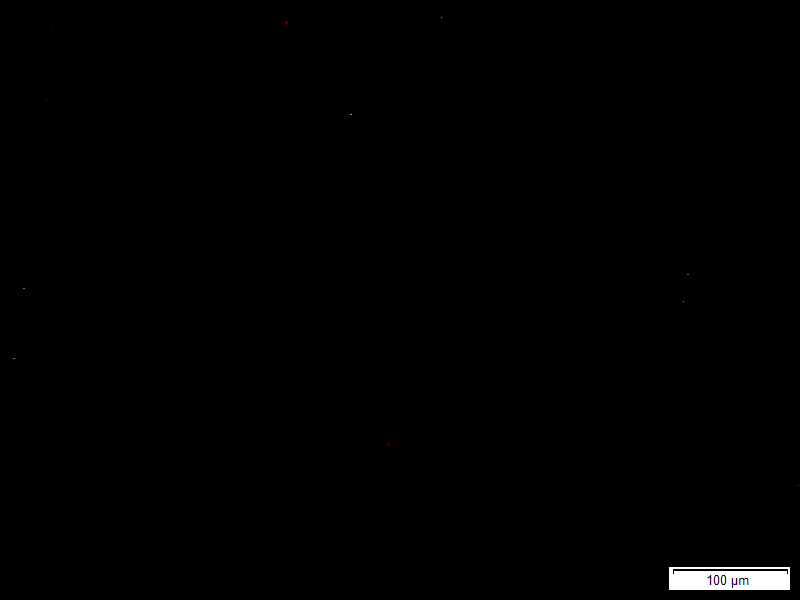

Supplement: S1 File — (ZIP) [file pone.0353062.s001.zip › S4 File. Fluorescent microscopy images of cell samples/beas-2b/Beas-2b muc 暗场.tif]

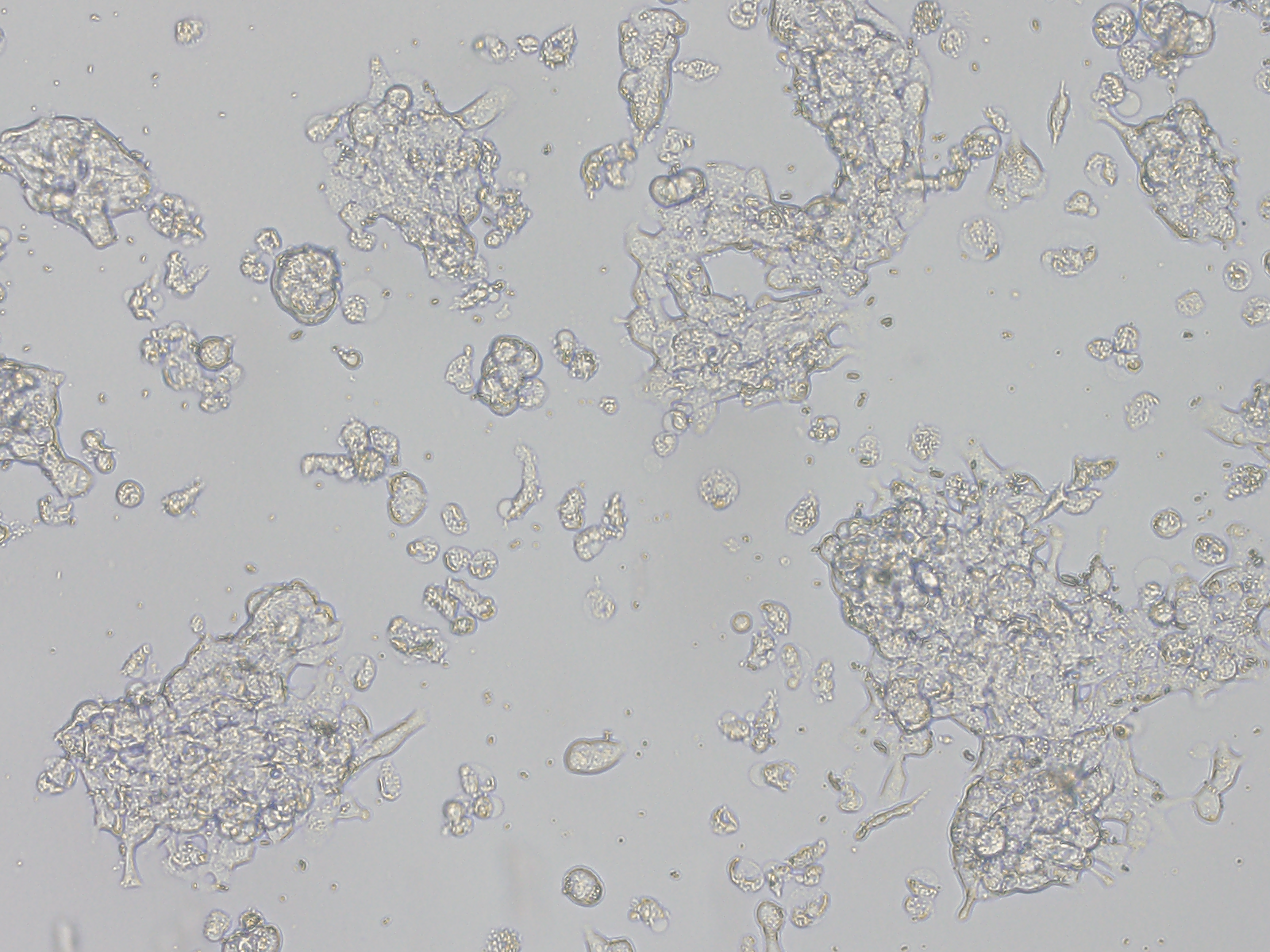

Supplement: S1 File — (ZIP) [file pone.0353062.s001.zip › S4 File. Fluorescent microscopy images of cell samples/ls174t/ls cmv 明.tif]

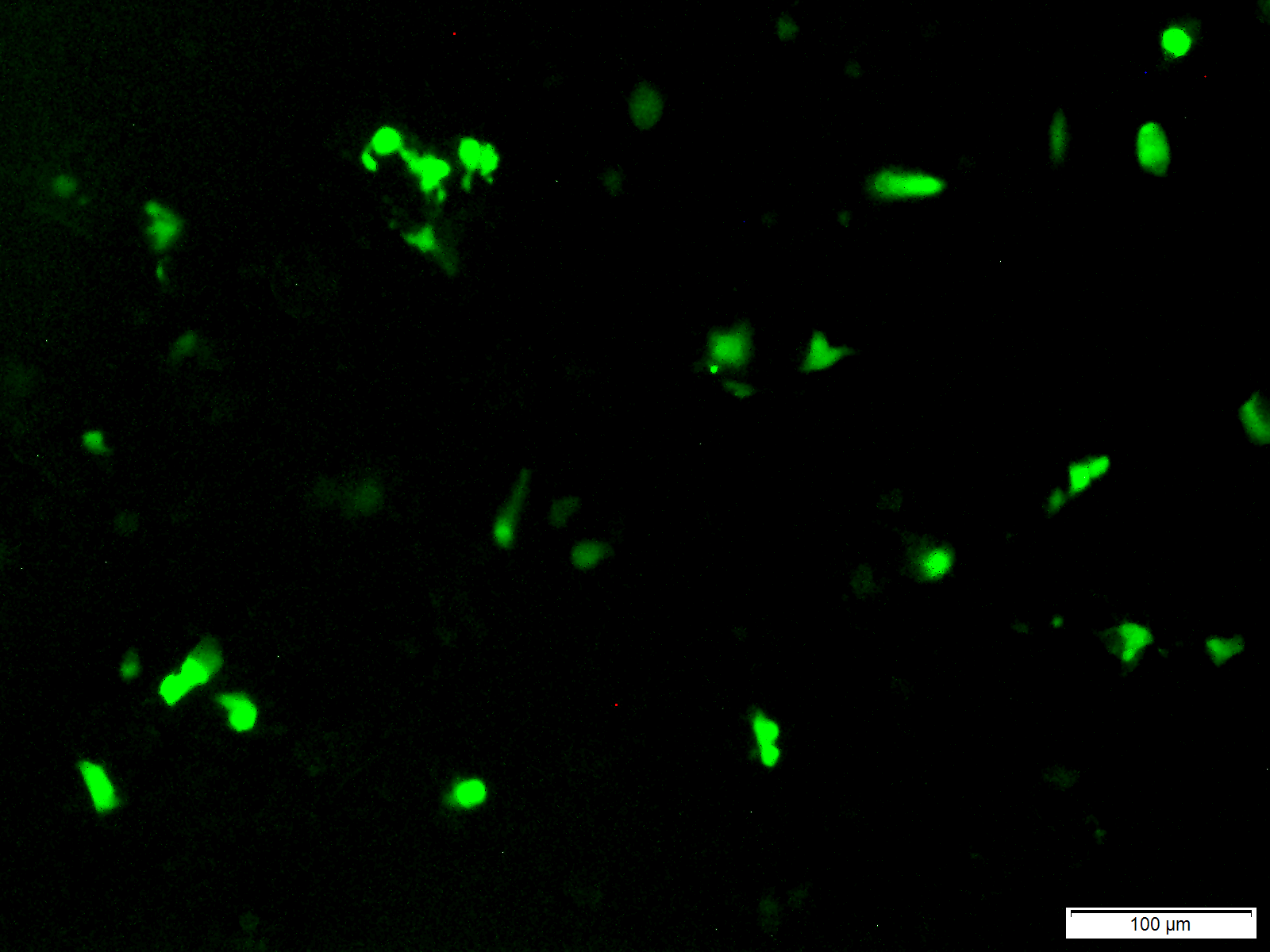

Supplement: S1 File — (ZIP) [file pone.0353062.s001.zip › S4 File. Fluorescent microscopy images of cell samples/ls174t/ls cmv 暗.tif]

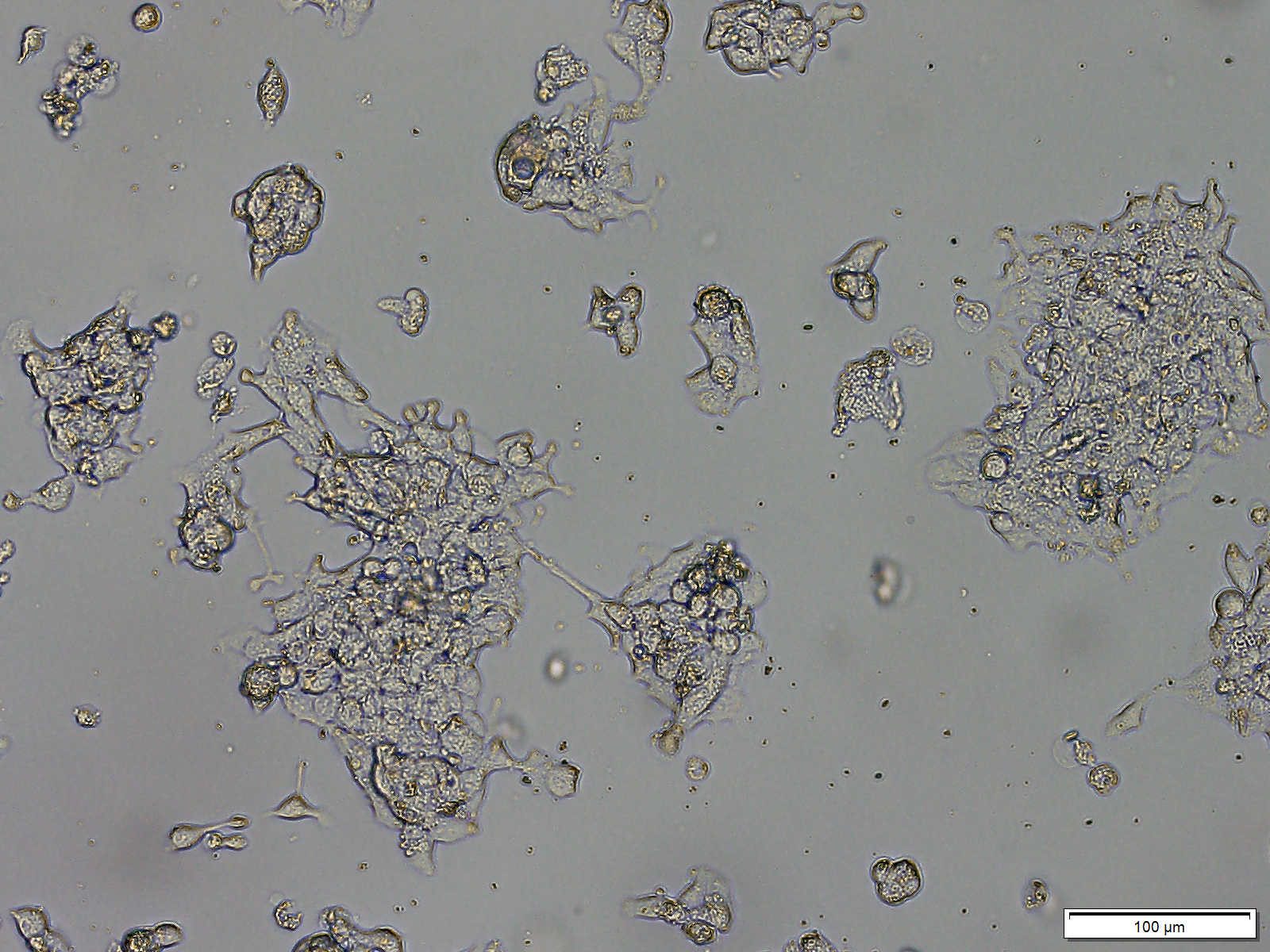

Supplement: S1 File — (ZIP) [file pone.0353062.s001.zip › S4 File. Fluorescent microscopy images of cell samples/ls174t/ls muc 明.tif]

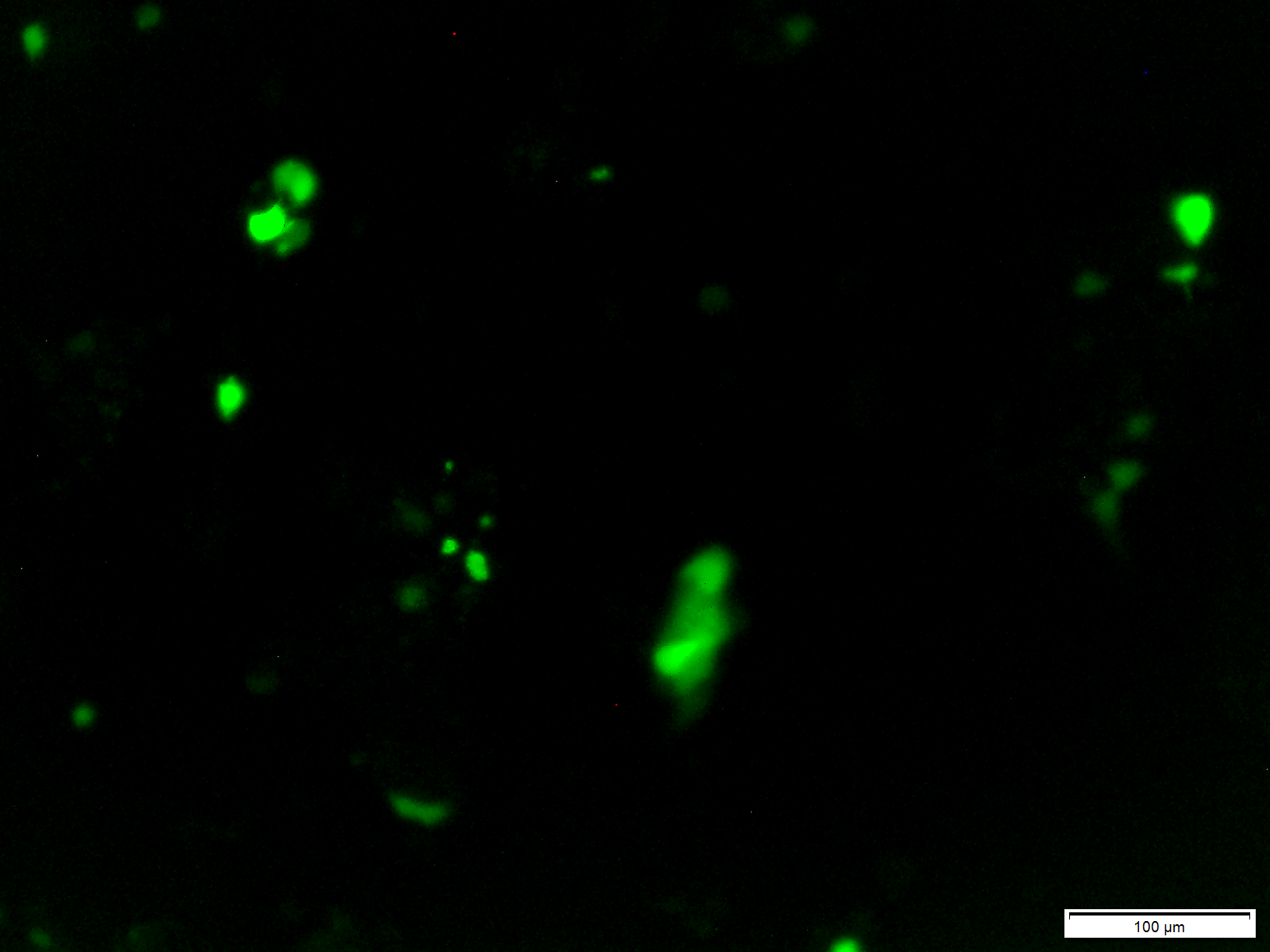

Supplement: S1 File — (ZIP) [file pone.0353062.s001.zip › S4 File. Fluorescent microscopy images of cell samples/ls174t/ls muc 暗.tif]

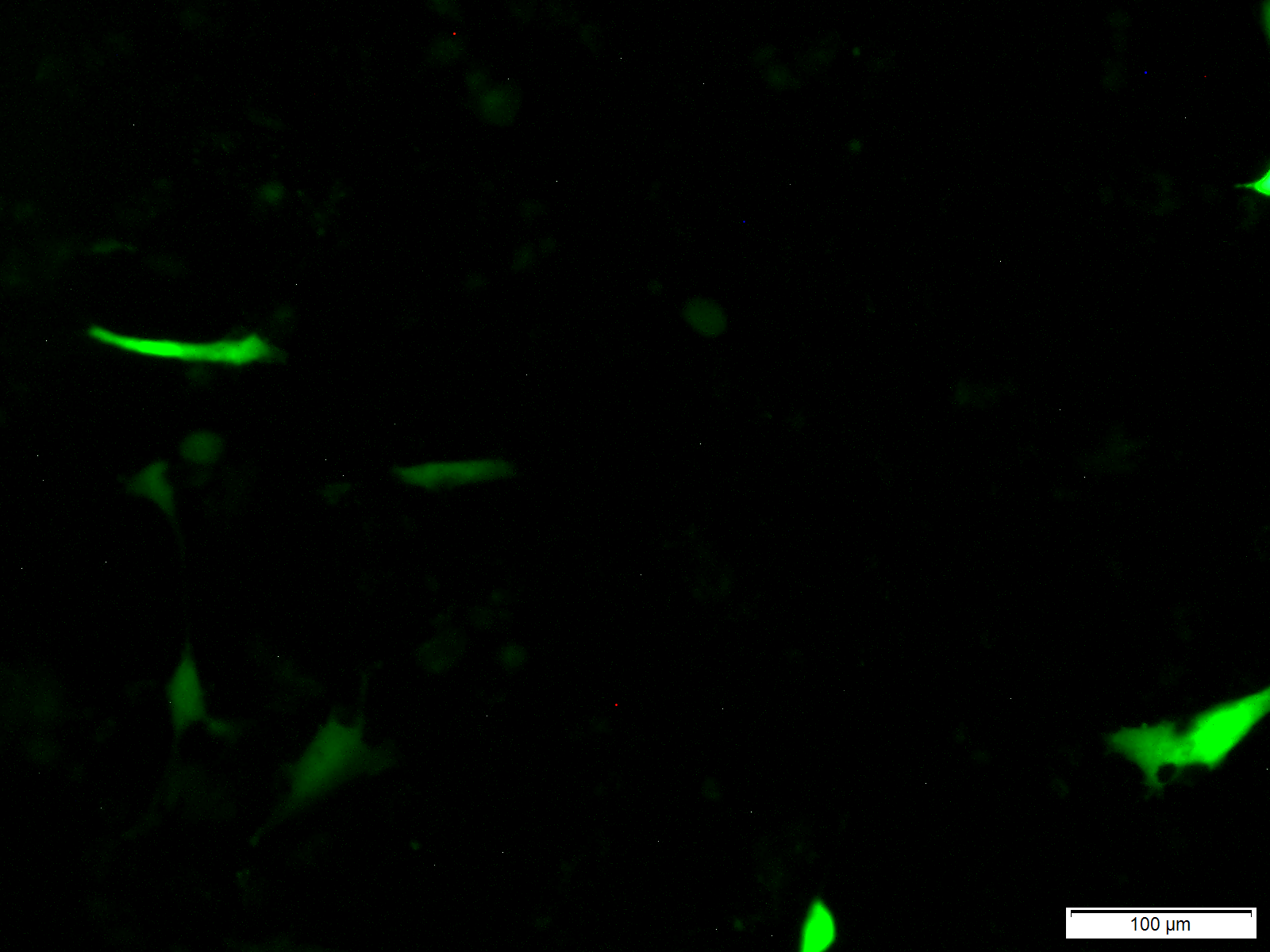

Supplement: S1 File — (ZIP) [file pone.0353062.s001.zip › S4 File. Fluorescent microscopy images of cell samples/lx2/lx2 cmv 暗.tif]

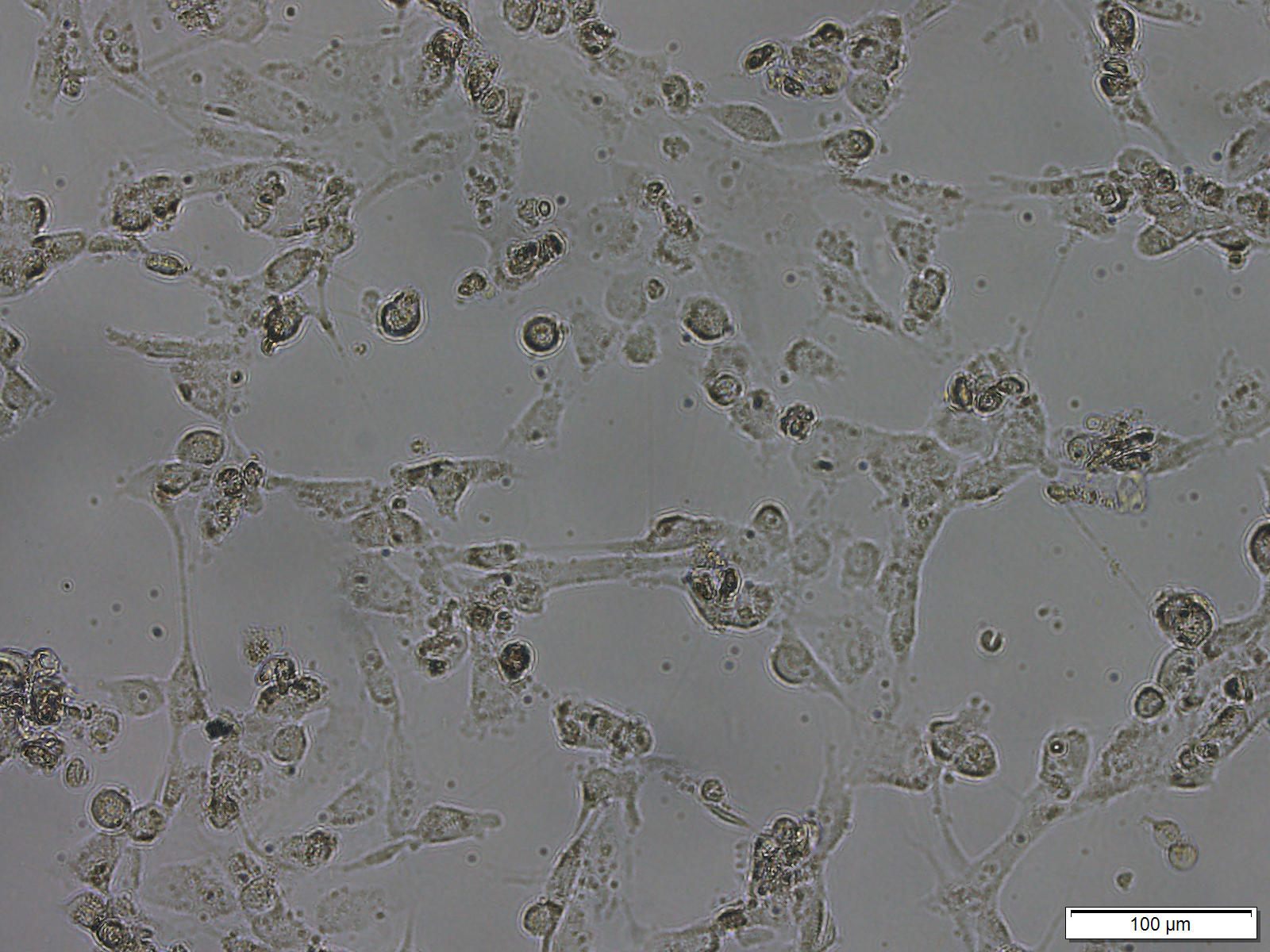

Supplement: S1 File — (ZIP) [file pone.0353062.s001.zip › S4 File. Fluorescent microscopy images of cell samples/lx2/lx2 cmv.tif]

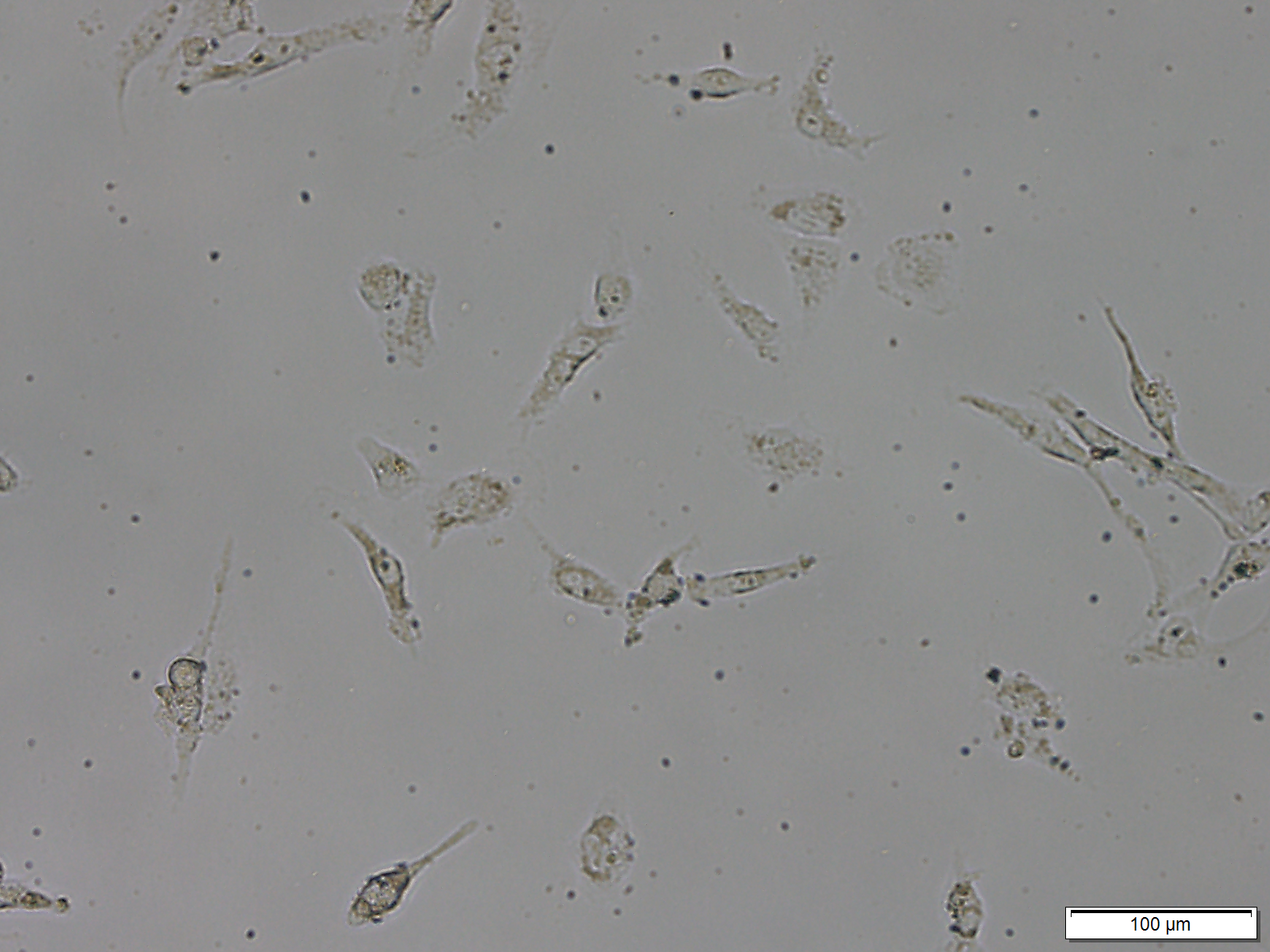

Supplement: S1 File — (ZIP) [file pone.0353062.s001.zip › S4 File. Fluorescent microscopy images of cell samples/lx2/lx2 muc.tif]

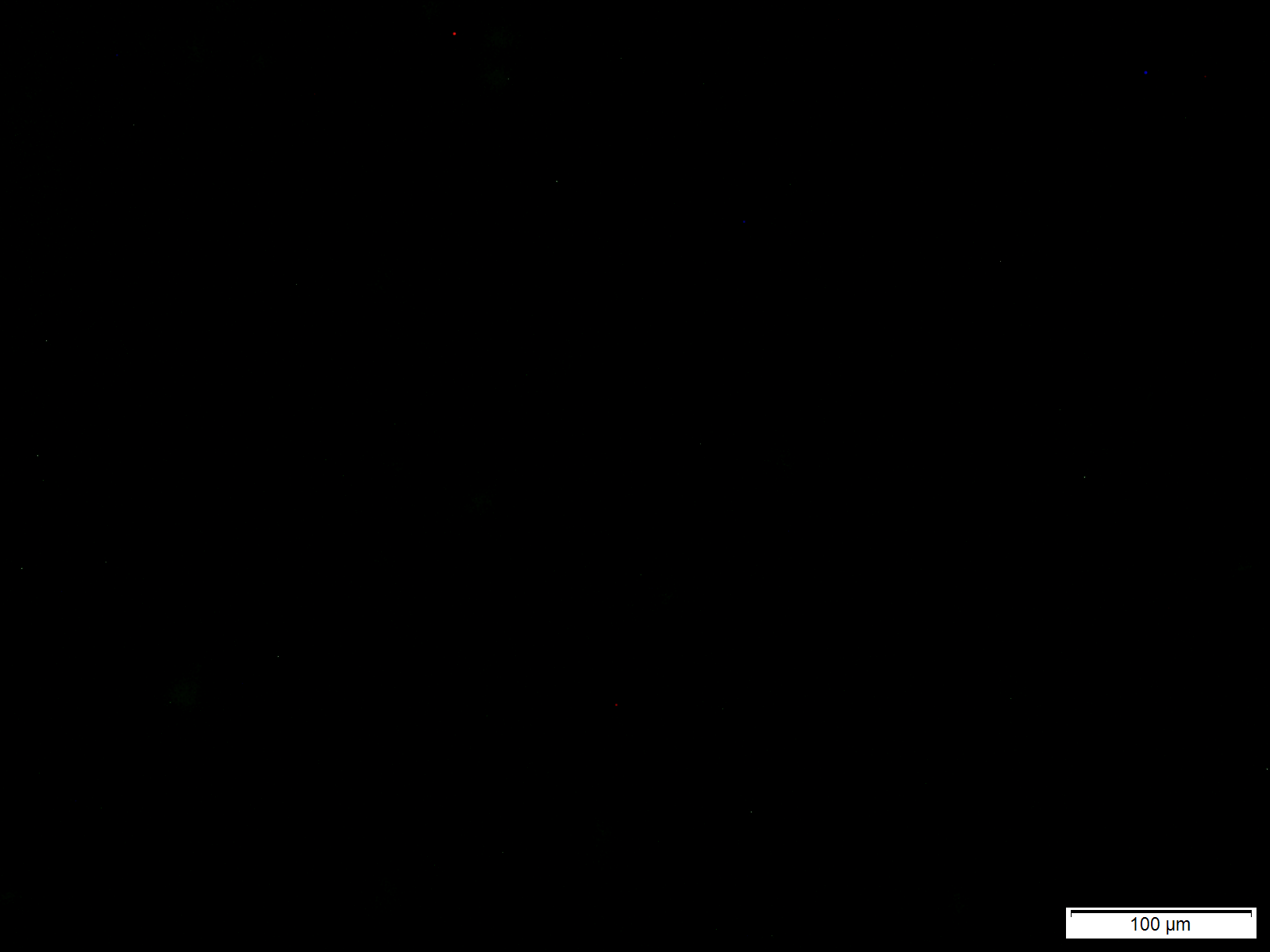

Supplement: S1 File — (ZIP) [file pone.0353062.s001.zip › S4 File. Fluorescent microscopy images of cell samples/lx2/lx2 muc暗场.tif]

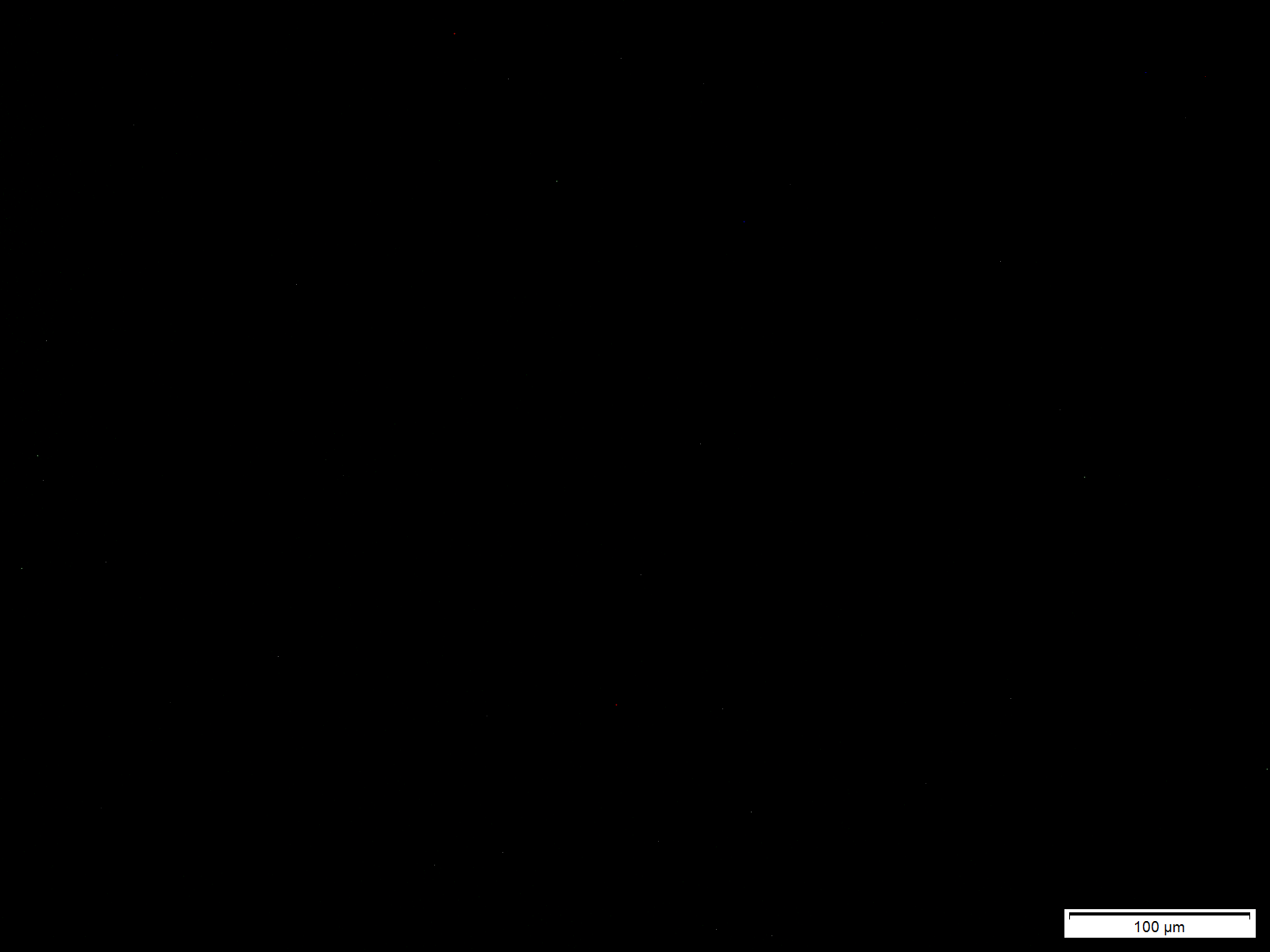

Supplement: S1 File — (ZIP) [file pone.0353062.s001.zip › S4 File. Fluorescent microscopy images of cell samples/u251/251 muc 暗场1.tif]

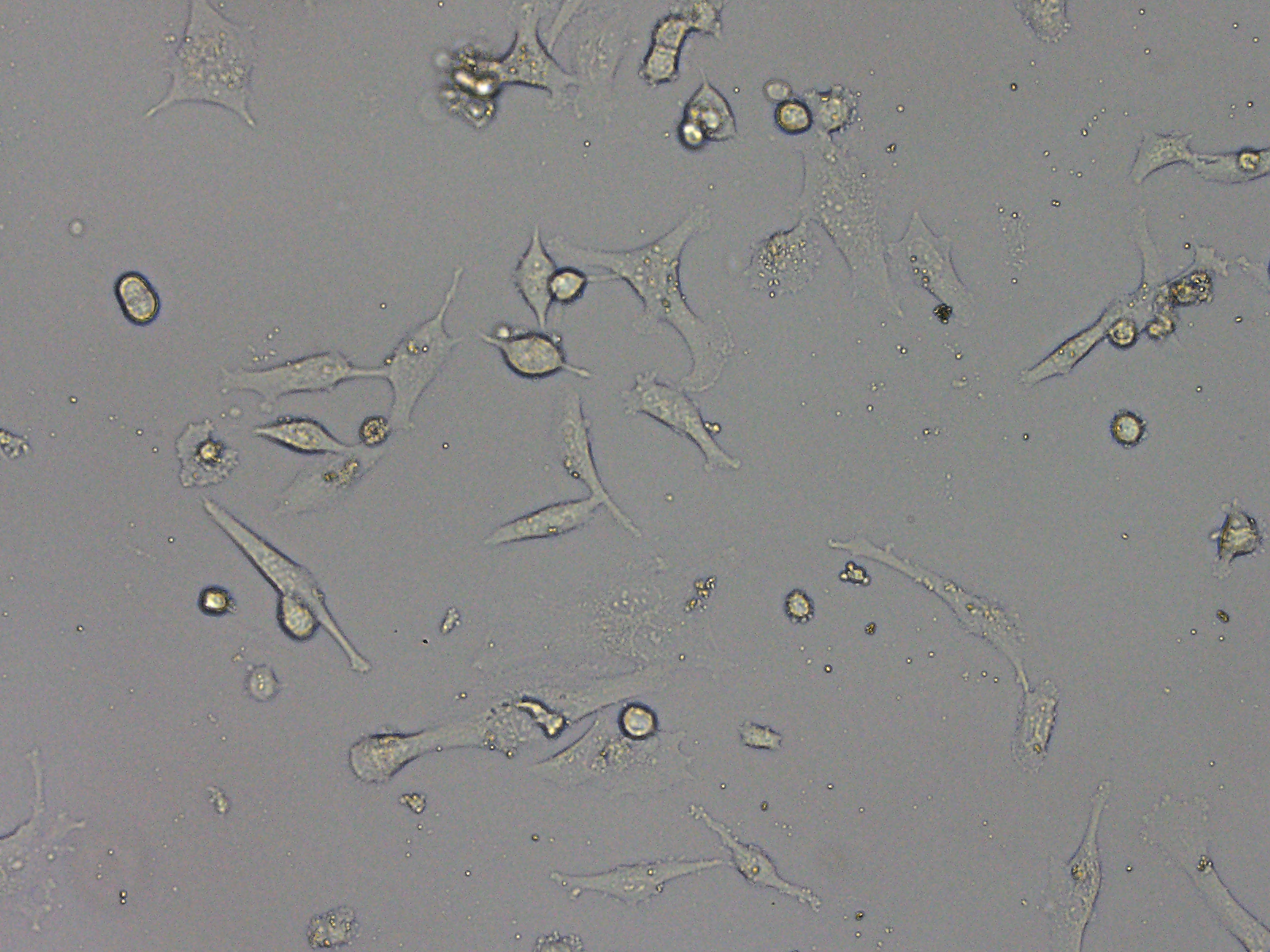

Supplement: S1 File — (ZIP) [file pone.0353062.s001.zip › S4 File. Fluorescent microscopy images of cell samples/u251/251 muc2.tif]

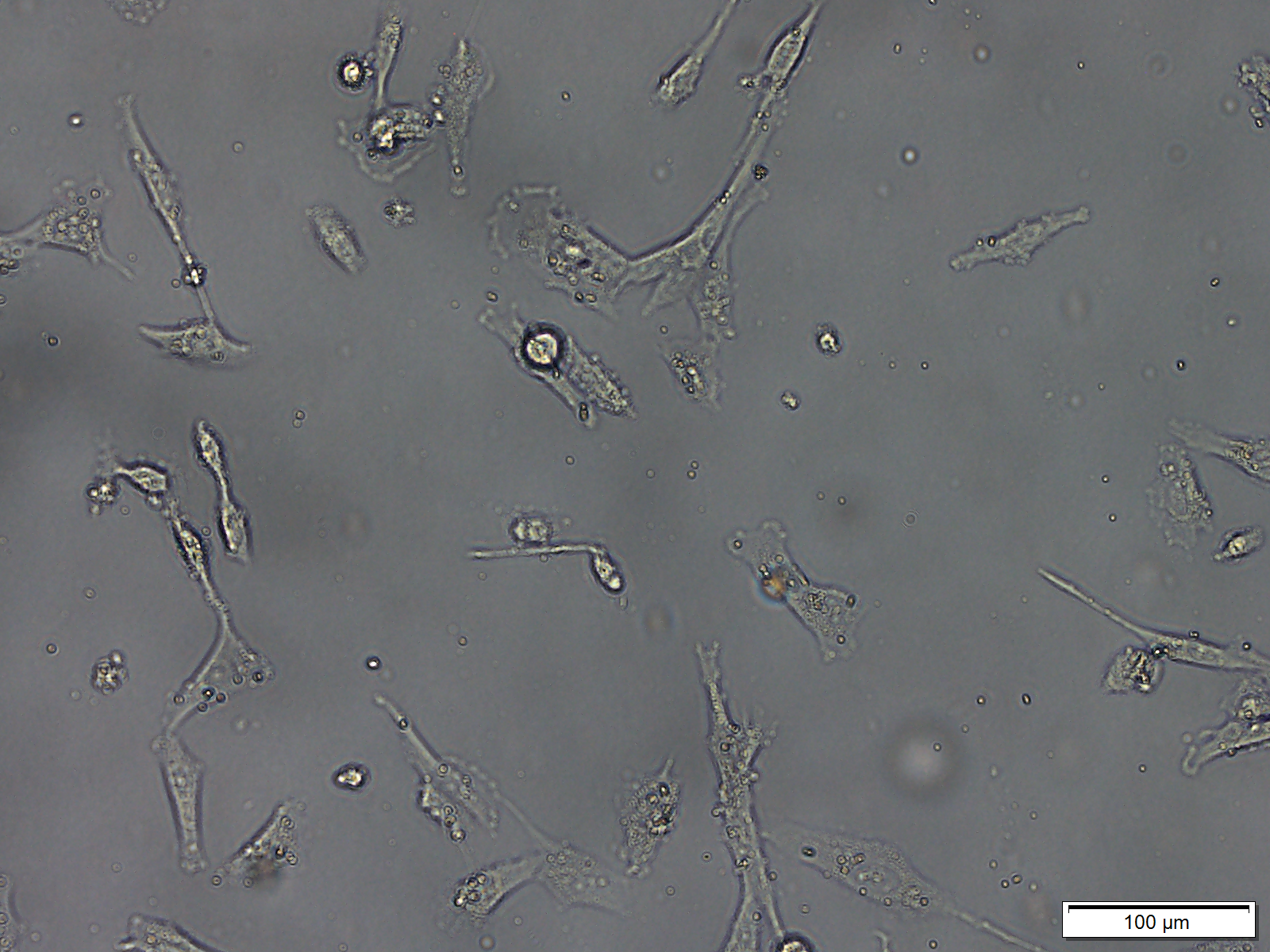

Supplement: S1 File — (ZIP) [file pone.0353062.s001.zip › S4 File. Fluorescent microscopy images of cell samples/u251/cmv 明场.tif]

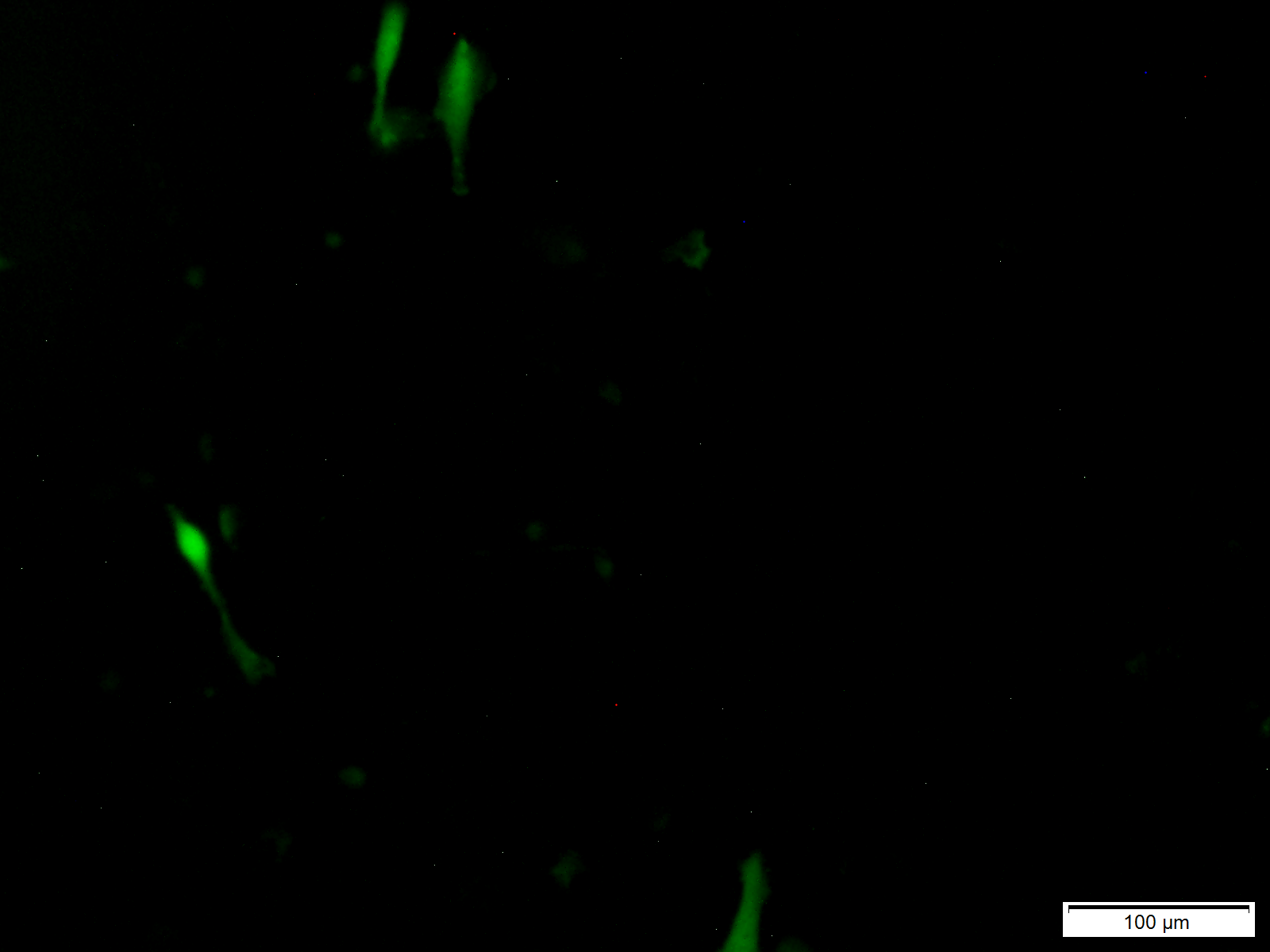

Supplement: S1 File — (ZIP) [file pone.0353062.s001.zip › S4 File. Fluorescent microscopy images of cell samples/u251/cmv暗场.tif]
